# Heterologous Expression and Evaluation of Novel *Plasmodium falciparum* Transmission Blocking Vaccine Candidates

**DOI:** 10.3389/fimmu.2022.909060

**Published:** 2022-06-23

**Authors:** Roos M. de Jong, Susheel K. Singh, Karina Teelen, Marga van de Vegte-Bolmer, Geert-Jan van Gemert, Will J. R. Stone, Emily Locke, Jordan Plieskatt, Michael Theisen, Teun Bousema, Matthijs M. Jore

**Affiliations:** ^1^ Department of Medical Microbiology, Radboud University Medical Center, Nijmegen, Netherlands; ^2^ Centre for Medical Parasitology at Department of Immunology and Microbiology, University of Copenhagen, Copenhagen, Denmark; ^3^ Department for Congenital Disorders, Statens Serum Institut, Copenhagen, Denmark; ^4^ Department of Immunology and Infection, London School of Hygiene and Tropical Medicine, London, United Kingdom; ^5^ PATH‘s Malaria Vaccine Initiative, Washington, DC, United States

**Keywords:** malaria, *plasmodium falciparum*, transmission blocking vaccines, recombinant expression, *drosophila melanogaster* S2 cells, *lactococcus lactis*

## Abstract

Malaria transmission blocking vaccines (TBV) aim to induce antibodies that can interrupt *Plasmodium falciparum* development in the mosquito midgut and thereby prevent onward malaria transmission. A limited number of TBV candidates have been identified and only three (Pfs25, Pfs230 and Pfs48/45) have entered clinical testing. While one of these candidates may emerge as a highly potent TBV candidate, it is premature to determine if they will generate sufficiently potent and sustained responses. It is therefore important to explore novel candidate antigens. We recently analyzed sera from naturally exposed individuals and found that the presence and/or intensity of antibodies against 12 novel putative surface expressed gametocyte antigens was associated with transmission reducing activity. In this study, protein fragments of these novel TBV candidates were designed and heterologously expressed in *Drosophila melanogaster* S2 cells and *Lactococcus lactis*. Eleven protein fragments, covering seven TBV candidates, were successfully produced. All tested antigens were recognized by antibodies from individuals living in malaria-endemic areas, indicating that native epitopes are present. All antigens induced antigen-specific antibody responses in mice. Two antigens induced antibodies that recognized a native protein in gametocyte extract, and antibodies elicited by four antigens recognized whole gametocytes. In particular, we found that antigen Pf3D7_0305300, a putative transporter, is abundantly expressed on the surface of gametocytes. However, none of the seven novel TBV candidates expressed here induced an antibody response that reduced parasite development in the mosquito midgut as assessed in the standard membrane feeding assay. Altogether, the antigen fragments used in this study did not prove to be promising transmission blocking vaccine constructs, but led to the identification of two gametocyte surface proteins that may provide new leads for studying gametocyte biology.

## Introduction

Malaria remains a major global health challenge with estimates of 241 million new cases and 627,000 malaria-related deaths in 2020 (1). The disease is caused by unicellular *Plasmodium* parasites which are transmitted by *Anopheles* mosquitoes. During the past two decades, there have been major investments in malaria control efforts, including an increased coverage of insecticide treated bed nets, faster diagnostics and improved treatment with artemisinin-based combination therapies (2). This has led to a steady decline of malaria morbidity and mortality till 2015, but since then incidence has plateaued and recently even increased (1). The success of the implemented interventions is threatened by emerging artemisinin and insecticide resistance. New effective tools, including the implementation of highly effective vaccines, will be needed to reach elimination goals (3).

Transmission of *Plasmodium* parasites through the population is dependent on the presence of gametocytes in peripheral blood of the human host and subsequent uptake by mosquitoes. Targeting this highly efficient transmission to mosquitoes is considered a crucial step in malaria elimination efforts. Transmission blocking vaccines aim to induce antibodies against surface proteins on sexual stage parasites, which act in the mosquito midgut by preventing sexual development and thereby interrupting parasite transmission. The three most advanced vaccine candidates (Pfs48/45, Pfs230 and Pfs25) were identified over 35 years ago as targets of functional antibodies in rodents immunized with crude sexual stage parasite extracts (4). These candidate transmission blocking vaccines (TBVs) have now reached early phase clinical testing (Ref (5–7). and clinicaltrials.gov: NCT04862416), however it is premature to determine if they will generate sufficiently potent and sustained responses.

Besides these vaccine candidates, several other sexual stage antigens have been identified, and analyzed in pre-clinical studies. Two of the better studied antigens are the gamete fusogen hapless 2 (HAP2) (8) and female gamete surface protein Pfs47 (9). Antibodies against specific epitopes of these antigens reduced transmission of *P. falciparum* parasites (10–14), supporting further investigation of these antigens as potential TBVs (15). Initiatives to identify (16) and characterize (17, 18) novel TBV candidates have been stimulated by the availability of sexual stage specific transcriptome (19) and proteome datasets (20, 21). These studies demonstrated that antigens enolase (17), Pf77 and PfMDV1 (18) induce antibodies with transmission reducing activity (TRA) in mice. However, future studies are needed to determine whether the observed efficacy is reproducible and should include the optimization of protein expression and vaccine formulations. These studies will be crucial to assess whether these antigens are indeed viable TBV candidates. Altogether, the number of vaccine candidates is limited, and new targets are needed to fill the (pre-)clinical pipeline in hope of providing vaccine candidates for clinical evaluation.

Polyclonal antibodies that are able to reduce parasite transmission in mosquito feeding assays have been observed in individuals who were naturally exposed to malaria (22). We previously performed immuno-profiling of antibodies from individuals with naturally acquired TRA and control individuals who did not develop TRA despite gametocyte exposure to identify the associated antigens (16). Antibody prevalence and intensity to Pfs48/45, Pfs230 and 43 other antigens were associated with TRA. Interestingly, almost half of the individuals with high TRA had no measurable antibodies against Pfs48/45 and Pfs230, indicating that the other 43 antigens may be involved in the induction of functional transmission reducing antibody responses in naturally infected individuals. Not all of these 43 hits were considered equally plausible TBV candidates; 12 novel putative surface expressed gametocyte antigens were considered high-priority antigens for evaluation as TBV candidates because of a known role in gamete viability or characteristics that suggest surface expression.

In this study, we designed protein fragments for these 12 antigens to evaluate whether these proteins are able to induce antibodies with TRA in mice. We used two expression systems that have been successful for expression of other *Plasmodium* surface antigens, including TBV candidates; *Drosophila melanogaster* S2 cells (23) and *Lactococcus lactis* (24). We analyzed the presence of native epitopes in recombinant proteins produced by these systems, by assessing reactivity of antibodies in malaria-endemic plasma samples. We subsequently induced antibodies in mice and analyzed whether the elicited antibodies showed reactivity with native parasite proteins by ELISA, western blot and immunofluorescent assays. Functionality of antibodies was assessed in the standard membrane feeding assays.

## Material and Methods

### Construct Design for Recombinant Protein Expression

A previous study 43 antigens with TRA-associated antibody responses, and generated a TBV candidate list selecting 13 antigens with a known role in gamete viability or characteristics that are compatible with surface expression (16). Potential surface expression was based on the predicted presence of transmembrane domains and/or a signal peptide, and predictions of the cellular component in which the protein functions (gene ontological terms). In the current study, we only included novel TBV candidates without prior information on functionality; Pf11.1 (Pf3D7_1038400) was excluded, because it has been previously shown that an α-Pf11.1 monoclonal antibody can block transmission (25). Gene fragments were designed for the remaining 12 proteins on the TBV candidate list.

Pf3D7_1449000 was expressed as full-length protein and only the endogenous signal peptide was removed. For expression of Pf3D7_1314500 we removed the signal peptide (26) and the C-terminal transmembrane domain predicted by TMHMM 2.0 (27, 28). For all other proteins (>100kDa), the fragment selected for expression included the fragment that showed a significant association with TRA in the protein microarray study (16). We subsequently selected a fragment without transmembrane helices predicted by TMHMM 2.0 (27, 28) and omitted low-complexity amino acid sequences. The secondary structure as predicted by JPRED V4 (29) was used to determine the fragment boundaries (i.e. not starting or ending in predicted α-helices or β-sheets). An overview of the protein fragments is shown in **Table 1** and **Supplementary Figure 1**. Gene sequences were based on the *Plasmodium falciparum* 3D7 genome [PlasmoDB.org (30)]. Two expression systems, *Drosophila melanogaster and Lactococcus lactis* were selected as protein expression systems based on prior experience.

**Table 1:** Overview of protein fragments and expression levels in *Drosophila melanogaster* S2 cells and *Lactococcus lactis*.

Gene ID	Gene description	Expression fragment (aa) for *D. melanogaster* and *L. lactis*	Study ID	Molecular Weight (kDa) of protein fragment	Expression in *D. melanogaster* S2 cells^&^	Expression in *L. lactis* ^&^	
						Without R0	With R0
PF3D7_0305300	Transporter, putative	1-413	TBC4	50.4	–	+	+
PF3D7_1014300	Conserved protein, unknown function	1080-1515	TBC5	51.3	–	–	+
PF3D7_1021100	Conserved Plasmodium protein, unknown function	1001-1607		73.5	–	–	–
PF3D7_1107900	Mechanosensitive ion channel protein	266-808		64.9	–	+-	+-
PF3D7_1143700	Conserved Plasmodium protein, unknown function	1-100	TBC1	13	+	+-	+
PF3D7_1306500	MORN repeat protein, putative	Fragment A: 811-1021		26.2	–	Excluded*	–
		Fragment B: 1187-1515		40.7	–	–	+-
		Fragment C: 1563-1689	TBC2	16.3	+	+	Excluded^#^
PF3D7_1314500	Transmembrane emp24 domain-containing protein	30-173	TBC6	17.9	+-	+	+
PF3D7_1324600	Conserved Plasmodium protein, unknown function	Fragment A: 663-945		33.2	–	–	–
		Fragment B: 1013-1465		55.5	+-	–	+-
PF3D7_1348000	Conserved Plasmodium protein, unknown function	1-112	TBC3	13.7	+	+	+
PF3D7_1360500	Guanylyl cyclase beta	Fragment A: 2154-2738		71.4	–	+-	–
		Fragment B: 2916-3179		31.8	+	–	–
PF3D7_1433200	Conserved Plasmodium protein, unknown	Fragment A: 1-535		65.7	–	–	+-
		Fragment B: 680-1001	TBC7	39.2	+	–	+
		Fragment C: 1002-1267		33.2	–	–	–
PF3D7_1449000	Gamete Egress and Sporozoite traversal protein	24-248		26.1	–	–	–

^&^Expression levels are based on small scale expression tests (**Supplementary Figure 1** and **Supplementary Tables 2, 3**). “-” not detected, “+-” low expression in soluble fraction, “+” soluble expression.

*Excluded due to the inability to generate a mutation-free expression plasmid.

^#^Excluded because antigen was already available from S2 cells and L. lactis.

### Protein Expression in *Drosophila melanogaster* S2 Cells

#### Plasmid Preparation and Generation of Stable Polyclonal *Drosophila melanogaster* Cell Lines

For expression in *Drosophila melanogaster* Schneider 2 (S2) cells, all genes were codon optimized and synthesized (GeneArt, Life Technologies). Predicted N-glycosylation sites were not changed. Inserts were sub-cloned using NgoMIV and NotI restriction enzyme sites into a modified pExpreS_2_-2 plasmid (ExpreS^2^ion Biotechnologies, Denmark) to include an N-terminal Kozak sequence (GCCACC), BiP insect signal peptide (MKLCILLAVVAFVGLSLG) and hexahistidine tag. All expression plasmids were verified by Sanger sequencing (Baseclear, the Netherlands). *D. melanogaster* S2 cells (ExpreS^2^ion Biotechnologies, Denmark) were transfected according to manufacturer’s instructions to generate stable polyclonal cell lines. Briefly, on day 0 cells were resuspended to 5 x 10^6^ cells/mL in EX-CELL420 medium (Sigma-Aldrich) supplemented with 10% fetal bovine serum (FBS). The following day, cells were resuspended to 2 x 10^6^ cells/mL EXCELL-420 medium without FBS and for each transfection 2 mL cell suspension was transferred to a T12.5 flask. 25 µL ExpreS^2^ Insect-TR 5x transfection reagent (ExpreS^2^ion Biotechnologies, Denmark) and 6.25 µg plasmid DNA was added followed by gently mixing. Flasks were incubated for 2 to 4 hours at 25°C before addition of 0.5 mL FBS. On day 2, geneticin (G418) was added at a final concentration of 4 mg/mL for selection. The cultures were maintained and expanded in EX-CELL420 medium supplemented with 10% FBS and 4 mg/mL geneticin for three weeks to establish stable polyclonal cell lines. Stably transfected cell lines were subsequently maintained in shake flasks at 25°C at 115 rpm in non-supplemented EX-CELL420. For protein expression, the cells were resuspended in fresh EX-CELL420 medium to 8 x 10^6^ cells/mL and supernatant was harvested five days after resuspension. Supernatants were stored at -20°C until further use.

#### Screening of Recombinant Protein Expression and Recombinant Protein Purification

For protein expression analysis of stably transfected S2 cells, 10 mL supernatant was incubated with 50 µL (50% suspension) of cOmplete™ His-tag resin (Roche). The resin was washed twice with phosphate buffer saline (PBS) and thrice with PBS with 5mM imidazole. The resin was mixed with 2x Tris-Glycine SDS sample buffer and heated for 5 minutes at 95°C to release the bound proteins. The samples were centrifuged, and the supernatant was analyzed by non-reducing SDS polyacrylamide gel electrophoresis (SDS-PAGE) followed by staining with Coomassie R-250 or western blotting. A titration of immunopurified R0.6C-His (31) was included to estimate protein quantities. Protein transfer to a 0.45 µm nitrocellulose membrane was performed using the Trans-Blot Turbo transfer system (Bio-Rad). An anti-His HRP conjugate kit (Qiagen) and ECL detection reagent (Amersham) were used for detection of His-tagged proteins. Blots were images on the ImageQuant™ LAS4000 (GE Healthcare).

Clarified five-day batch culture (100-500 mL) supernatant was filtered and directly applied on a cOmplete™ His-Tag purification column (Roche). Bound protein was eluted using a gradient elution with imidazole in PBS (pH7.3). Peak fractions were pooled, and imidazole was removed through overnight dialysis 4°C against PBS at using dialysis membrane tubing (Spectra/Por^®^). The dialyzed sample was concentrated using an Amicon^®^ Ultra centrifugal concentrator (Merck) before proteins were separated by size exclusion chromatography using a Superdex 200 Increase 10/300 GL column (GE Healthcare). Fractions were analyzed on non-reducing SDS-PAGE gels stained with Coomassie R-250. Fractions were pooled based on protein quantity and purity. The pooled fractions were subsequently concentrated using an Amicon^®^ Ultra centrifugal concentrator (Merck) until a final concentration of 0.5-1 mg/mL was reached as determined by NanoDrop1000 (Thermo Scientific). Protein samples were filter-sterilized, snap-frozen in liquid nitrogen and stored at -80°C until further use. For purification of proteins that appeared to have low solubility during test purification, the detergent Empigen^®^ BB (Sigma-Aldrich) was added to all buffers at a final concentration of 0.2%.

### Protein Expression in *Lactococcus lactis*

#### Plasmid Preparation

For expression in *Lactococcus lactis*, the majority of gene fragments were amplified from *P. falciparum* NF54 cDNA. Total extracted nucleic acids (MagNA Pure, Roche) were treated with DNaseI treated (Promega) and used for cDNA synthesis using the High Capacity cDNA synthesis kit (Applied Biosystems™). Gene fragments were amplified from cDNA using PrimeSTAR GLX polymerase (Takara bio) according to manufacturer’s instructions. The primers used for amplification are listed in **Supplementary Table 1**. Amplicons were cloned in plasmid pSS1 using SalI and BglII or BamHI restriction sites (32). For expression as fusion with R0, the N-terminal part (79-1500bp) of GLURP, fragments were subcloned in plasmid pSS2 using SalI and BglII or BamHI restriction sites (32). The gene fragments for R0 fusions with Pf3D7_1107900, Pf3D7_1306500A and Pf3D7_1324600A were codon optimized and synthesized by GeneArt (Life technologies) due to difficulties to obtain mutation-free PCR products from cDNA.

#### Transformation, Screening and Recombinant Protein Purification

Plasmids were transformed into *L. lactis* MG1363 by electroporation as described (33) and randomly five single colonies from each transformation were picked and grown overnight at 30°C in 5 mL lactic acid bacteria (LAB) medium supplemented with 4% (w/v) glycerol-phosphate and 5 µg/mL erythromycin (34). Protein expression in clarified supernatant was analyzed by ELISA using anti-His-HRP C-term (Miltenyi Biotec). One clone of each transformation that showed good protein expression was inoculated for 0.75-1L fermentation in LAB medium supplemented with redox coupling agents cysteine (5mM) and cystine (0.5mM). After 3 hours of growth, the pH was reduced to 6.4 ± 0.1 and this level was maintained by a pH-controlled supplementation of 2M NaOH for 8 hours until cell density reached an OD_600_ value of approximately 11. Proteins were purified from clarified supernatants using a HisTrap HP columns (GE Healthcare) as described (32). Pooled fractions containing the protein of interest were kept at -80°C until further use. The pooled fractions were buffer exchanged to PBS and concentrated using an Amicon^®^ Ultra centrifugal concentrator (Merck) before size exclusion chromatography using a Superdex 200 Increase 10/300 GL column (GE Healthcare). Fractions were analyzed by non-reducing SDS-PAGE gels stained with Coomassie R-250. Fractions were pooled based on monomeric protein concentration and the ratio between protein of interest and host cell contaminants. The pooled fractions were subsequently concentrated with an Amicon^®^ Ultra centrifugal concentrator (Merck) until a final concentration above 0.5 mg/mL was reached as determined by NanoDrop1000 (Thermo Scientific). Protein samples were filter sterilized, snap-frozen in liquid nitrogen and stored at -80°C until further use.

### Recombinant Protein Analysis

Purified recombinant proteins from both expression systems were mixed with 4x NuPAGE™ sample buffer (Thermo Fisher). A final concentration of 10mM dithiothreitol was added to the sample buffer to reduce samples. Samples were heated for 10 minutes at 70°C and separated on a 4-20% SurePAGE™ Bis-Tris gel (GenScript) alongside a Precision Plus Dual Color protein marker (Bio-Rad). Total protein was visualized by staining the gel directly with InstantBlue™ protein stain (Abcam). Glycosylation of proteins was analyzed by staining the gel with the Pierce™ Glycoprotein Staining kit (Thermo Scientific). The amino acid sequences of the successfully produced proteins are presented in **Supplementary Table 2**.

### Mouse Immunogenicity Studies

Per antigen, we immunized a group of 5 female CD-1 mice (Charles Rivers, Germany). Antigens were formulated in Montanide™ ISA720 (SEPPIC, France) following manufacturer’s instructions and injected subcutaneously. We immunized mice three times at three week intervals; each mouse receiving 10 µg per injection. We sacrificed animals for blood collection two weeks after the last immunization. A positive control group of 5 mice was immunized following the same protocol, receiving 10 µg R0.6C per injection; R0.6C is an antigen that previously yielded functional activity (31). All animal procedures complied with national regulations and were approved by the ethics committee of the Radboud University Medical Center.

### Enzyme-Linked Immunosorbent Assays (ELISA)

Immunoreactivity of antibodies in endemic plasma to the recombinant proteins was analyzed by antigen ELISA. Nunc MaxiSorp™ 96-wells plates (Thermo Fisher) were coated overnight at 4°C with 1 µg/mL recombinant antigen in PBS. Only antigens without R0 were used in this assay, due to the likely interference by GLURP antibodies present in endemic plasma. Plates were blocked with 5% skimmed milk in PBS and subsequently incubated with 1:200 plasma diluted in 1% skimmed milk in PBS with 0.05% Tween-20 (mPBST). Forty endemic plasma samples from Dutch expatriates and Africans, and seven negative control (Dutch donors) samples were included. Plates were subsequently incubated with 1:40,000 Anti-Human IgG HRP (Pierce™). Plates were washed before adding 100 µL tetramethylbenzidine. The color reaction was stopped by adding 50 µL 0.2M H_2_SO_4_ and the optical density was read at 450 nm on an iMark™ microplate absorbance reader (Bio-Rad).

To assess antibody responses in mouse sera, Nunc MaxiSorp™ 96-wells plates (Thermo Fisher) were coated overnight at 4°C with 0.2-1 µg/mL recombinant antigen in PBS. Plates were blocked with 5% skimmed milk in PBS and subsequently incubated with a 3-fold serial dilution (starting at 1:100) of individual mice sera in mPBST. After washing, the plates were incubated with 1:3,000 polyclonal rabbit anti-Mouse IgG HRP (DAKO, Germany). Development of ELISAs was performed as described above. Antibody midpoint titers (EC50) were calculated by sigmoidal curve fitting using GraphPad Prism 5 (GraphPad Software).

Native antigen recognition by mouse sera was assessed in gametocyte ELISA. *P. falciparum* NF54 gametocyte extract was prepared as described previously (34). Nunc MaxiSorp™ 96-wells plates (Thermo Fisher) were coated overnight at 4°C with lysate equivalent to 75,000 gametocytes per well. Plates were blocked with 5% skimmed milk in PBS and subsequently incubated with a 1:100 dilution of individual mice serum in mPBST. After washing, plates were incubated with 1:3,000 dilution polyclonal rabbit anti-Mouse IgG HRP (DAKO, Germany). Development of ELISAs was performed as described above.

### Gametocyte Extract Western Blot

*P. falciparum* NF54 gametocyte extract was prepared as described previously (34). The extract was mixed with 4x NuPAGE™ LDS sample buffer and heated for 10 minutes at 70°C before loading on a 4-12% Bis-Tris 2D-well gel. Precision Plus Dual Color protein marker (Bio-Rad) was used as size standard. Proteins were transferred to a 0.45 µm nitrocellulose membrane using the Trans-Blot Turbo transfer system (Bio-Rad). The blot was blocked with 5% skimmed milk in PBS and cut into strips. Each strip was incubated with a 1:100 dilution of pooled final bleed mice serum. After washing, the strips were incubated with 1:10,000 Goat Anti-Mouse IRDye 680RD (Li-cor, Cat. No. 926-68070). One strip was incubated with 1:100 polyclonal serum from rabbits immunized with R0. This strip was subsequently incubated with 1:10,000 Goat Anti-Rabbit IRDye 800CW (Li-cor, Cat. No. 926-32211). Strips were imaged using the Odyssey^®^ CLx system (Li-cor).

### Immunofluorescence Assays (IFAs)

Recognition of native antigens was also assessed in IFAs with whole gametocytes and gametes. For gametocyte IFAs, purified *P. falciparum* NF54 gametocytes were spotted on poly-lysine coated slides, fixed with 4% paraformaldehyde and half of the slides were permeabilized with 0.1% Triton X-100 in PBS. Gametocytes were incubated with a 1:100 dilution of pooled final bleed mice sera. Slides were subsequently stained with 1:1,000 Alexa Fluor^®^ 488 Chicken Anti-Mouse IgG (H+L) (Invitrogen, Cat. No. A21200). Slides were mounted with Vectashield^®^ (Vector Laboratories) before imaging.

In order to assess reactivity with female gametes, N-acetyl glucosamine treated *P. falciparum* NF54 gametocytes were activated by incubation in fetal calf serum (FCS) for one hour at room temperature. Female gametes were stained in solution with a 1:20 dilution of pooled final bleed mice sera. They were subsequently incubated with 1:200 Alexa Fluor^®^ 488 Chicken Anti-Mouse IgG (H+L) (Invitrogen, Cat. No. A21200). Parasites were imaged with an AxioCam ICc1 fitted on a Axio Scope.A1 microscope (Zeiss, Germany).

### IgG Purification

Serum pools of 600 µL for each mice group (~120 µL per mouse) were prepared and IgGs were purified using AB Spintrap™ columns (GE Healthcare). For TBC3^DM^ and TBC3^LL^, sera from one mouse and for TBC2^DM^ sera from two mice were excluded from the serum pool due to low antigen-specific antibody titers. A buffer exchange to milli-Q was performed using Amicon^®^ Ultra 30k centrifugal concentrators (Merck).

### Standard Membrane Feeding Assay (SMFAs)

SMFAs were performed as described previously (35). Briefly, *Anopheles stephensi* mosquitoes from a colony maintained at Radboudumc (Nijmegen, NL), were fed with mature *P. falciparum* NF54 gametocyte cultures mixed with purified IgGs diluted in FCS and active human complement. Mosquito midguts from 20 mosquitoes were typically dissected per condition, stained with mercurochrome and checked for the presence of oocysts 6-8 days after the blood meal. Occasionally, fewer mosquitoes were available for examination due to lower feeding or survival rates. Transmission reducing activity was calculated as a reduction in oocyst intensity (oocysts per mosquito midgut) compared to a negative control in which no test IgG (FCS control) was added to the blood meal. A negative binomial model was used to calculate the transmission reducing activity 100 x (1 exp(*β*)) where *β* is the estimated regression coefficient for intervention effect, and significance of TRA (β ≠ 0) was determined using a Z test statistic. These statistical analyses were done in R (version 4.1.2).

## Results

### Recombinant Protein Expression of Novel TBV Candidates in *Drosophila melanogaster* S2 Cells

**Figure 1:**
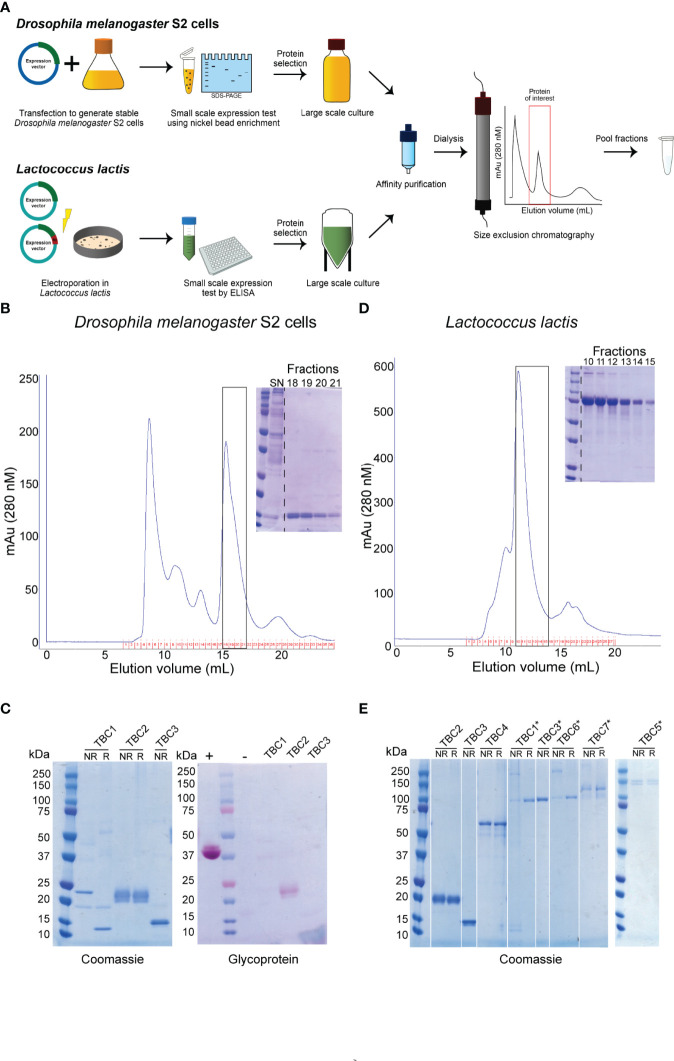
Overview of recombinant protein purification and analysis. **(A)** Illustration of the expression and purification strategy of recombinant proteins. Representative purifications on a Superdex 200 10/300 GL of a nickel-affinity purified protein expressed in **(B)** *Drosophila melanogaster* S2 cells or **(D)** *Lactococcus lactis.* Inset: Coomassie-stained non-reducing polyacrylamide gel of monomeric peak fractions. The purified proteins expressed in *Drosophila melanogaster* S2 cells **(C)** and *Lactococcus lactis* **(E)** were ran non-reduced (NR) and reduced (R) on a polyacrylamide gel and analyzed for purity using Coomassie-staining. Proteins expressed in S2 cells were also analyzed by glycoprotein staining **(C)**. *indicate antigens that were expressed as R0 chimeras. + and – indicate positive and negative controls, respectively. All antigens expressed as R0 chimeras run at a higher molecular weight, due to the glutamate content of R0.

We aimed to express a set of *P. falciparum* sexual stage antigens that have been associated with TRA responses in naturally infected individuals and were selected as plausible TBV candidates based on evidence for surface expression and/or a role in gamete viability (16). We designed plasmids for the expression of 18 protein fragments from 12 plausible TBV candidates and generated stable transfected *D. melanogaster* S2 cell lines for all 18 fragments (**Figure 1A** and **Table 1**). Analysis of the supernatant showed clear expression for 5 out of 18 antigens as assessed by western blot (**Supplementary Figure 2**). His-tagged proteins were purified directly from the supernatant using a two-step method (**Figures 1A, B**). For all antigens except 1306500C (TBC2^DM)^, we observed aggregation during size exclusion chromatography (**Supplementary Figure 3**). Addition of the detergent Empigen^®^ BB reduced protein aggregation and increased overall purity (**Supplementary Figure 3**). Antigens 1360500B and 1433200B could not be purified in sufficient yield and quantity, while 1143700 (TBC1^DM^), 1306500C (TBC2^DM^) and 1348000 (TBC3^DM^) were successfully purified for immunization studies.

The three purified proteins were of high purity as analyzed by Coomassie-staining of polyacrylamide gels (**Figure 1C**). TBC2^DM^ and TBC3^DM^ run at their expected molecular weight, while TBC1^DM^ appears to form a dimer through disulphide bridges. TBC2^DM^ is glycosylated, while the two other proteins lack glycosylation.

### Recombinant Protein Expression of Novel TBV Candidates in *Lactococcus lactis*

We also aimed to express all antigens in *L. lactis* to expand the number of recombinant proteins available for mouse immunizations (**Figure 1A** and **Table 1**). We had to exclude the expression of antigen 1306500A, because we were not able to generate a mutation-free expression plasmid. Seven proteins were expressed at substantial levels (OD>0.2) in the supernatant of at least one transformant as analyzed by ELISA (**Supplementary Table 3**). For each of these proteins, the highest expressing clone was selected for fermentation and His-tagged proteins were purified using a two-step purification method (**Figures 1A, D**). While the yield for four antigens was too low for immunization studies, antigens 1306500C (TBC2^LL^), 1348000 (TBC3^LL^) and 0305300 (TBC4^LL^) were obtained in sufficient quantity and purity for further evaluation.

Fusion of the N-terminal R0 domain of the glutamate-rich protein (GLURP) to the Pfs48/45 fragment 10C has shown to greatly improve expression levels of correctly folded 10C protein in *L. lactis* (34). We therefore also expressed the antigens in *L. lactis* as R0 fusion protein in order to further increase the number of antigens available for immunization studies. Fermentations were performed for 9 chimeric antigens that showed expression in screening experiments (**Supplementary Table 4**). R0.0305300 was excluded due to extensive multimerization and the protein yield of the antigens R0.1107900, R0.1324600A and R0.1433200A was too low to proceed. The protein fragments R0.1143700 (TBC1^LL*^), R0.1348000 (TBC3^LL*^), R0.1014300 (TBC5^LL*^), R0.1314500 (TBC6^LL*^), and R0.1433200B (TBC7^LL*^) were successfully purified. They were of high purity with some multimerization as analyzed by Coomassie-staining of polyacrylamide gels (**Figure 1E**) and used for further evaluation.

### Recombinant *P. falciparum* Proteins Contain Native Epitopes

**Figure 2:**
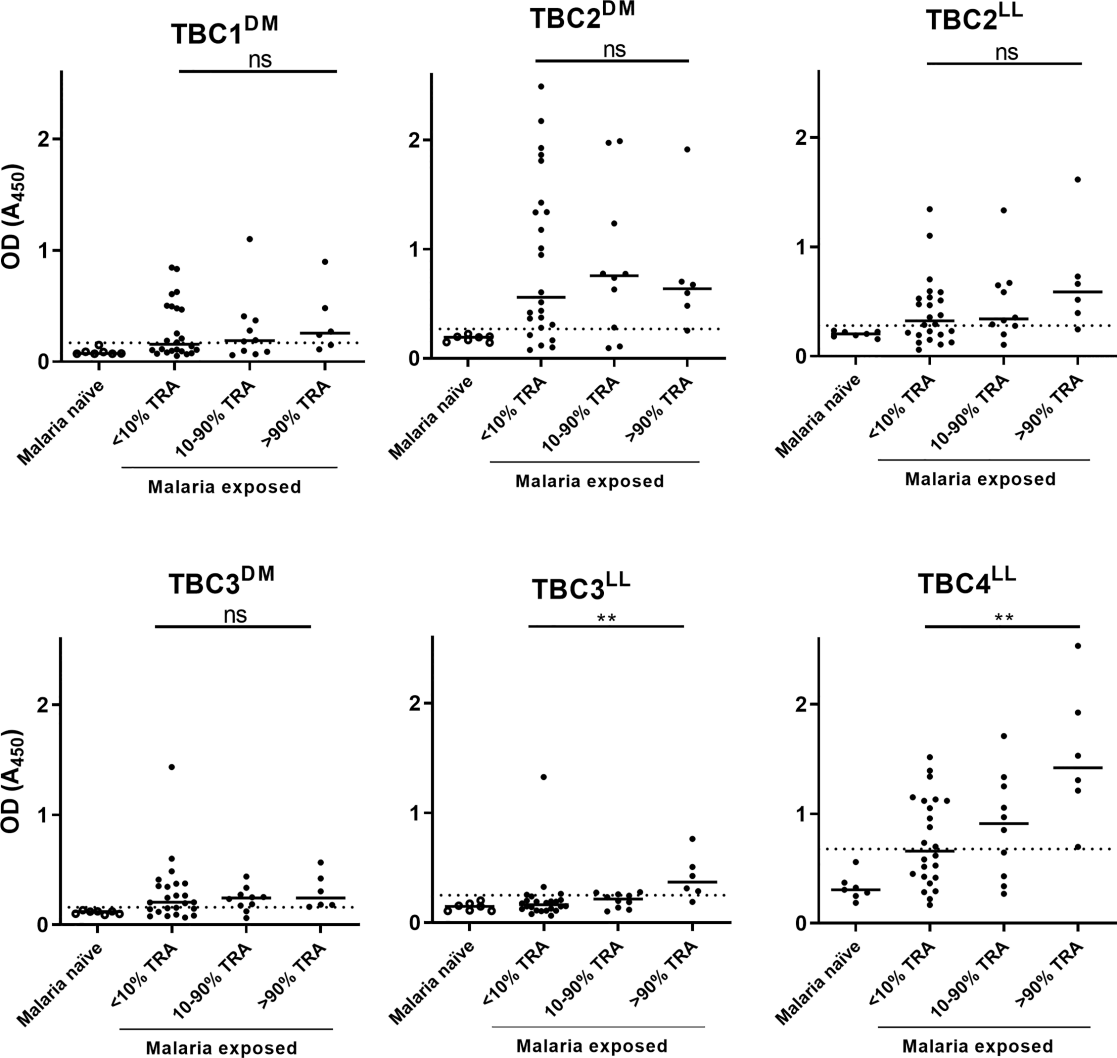
Recombinant proteins show immunoreactivity with plasma samples from individuals living in malaria-endemic areas. Forty malaria-endemic and 7 malaria-naïve plasma samples were analyzed in antigen ELISAs against the respective immunogen indicated. Malaria-endemic samples are categorized based on their transmission reducing activity (TRA) as determined by Stone et al. (16). Optical density (OD) values are reported as individual raw absorbance measurements at 450nm with the median shown as bar. The dotted line represents the mean + 3*SD of the malaria-naïve plasma samples. Samples with OD values above this cut-off are considered positive. DM: *Drosophila melanogaster*, LL: *Lactococcus lactis*. Comparison between the <10% and >90% TRA samples was performed using the Mann-Whitney test (**<0.005, ns, not significant).

The presence of native epitopes in the recombinant proteins was assessed by analyzing reactivity of antibodies in plasma samples from malaria-exposed individuals. Only the 6 antigens that were expressed without R0 were tested, because of the likely presence of α-R0 antibodies in endemic plasma (36). We selected 40 malaria-endemic plasma samples that have previously been used to determine TRA-associated antibody responses (16) and had sufficient material available for ELISA. Purified IgGs from these plasma samples had low (<10% TRA), medium (10-90%) or high (>90%) TRA (16) and results were presented in these bins. Additionally, we included plasma samples from 7 malaria-naïve controls to establish cutoffs for seropositivity. Antigen recognition by naturally induced antibodies was observed for all 6 antigens, indicating the presence of native epitopes in the recombinantly produced antigens (**Figure 2**). Despite a small number of available samples with high levels of TRA (>90% TRA, n=6), antibody responses against TBC3^LL^ (p=0.0026) and TBC4^LL^ (p=0.0034) were statistically significantly higher in the samples with >90% TRA compared to the samples with <10% TRA.

### Immunogenicity in Mice

**Figure 3:**
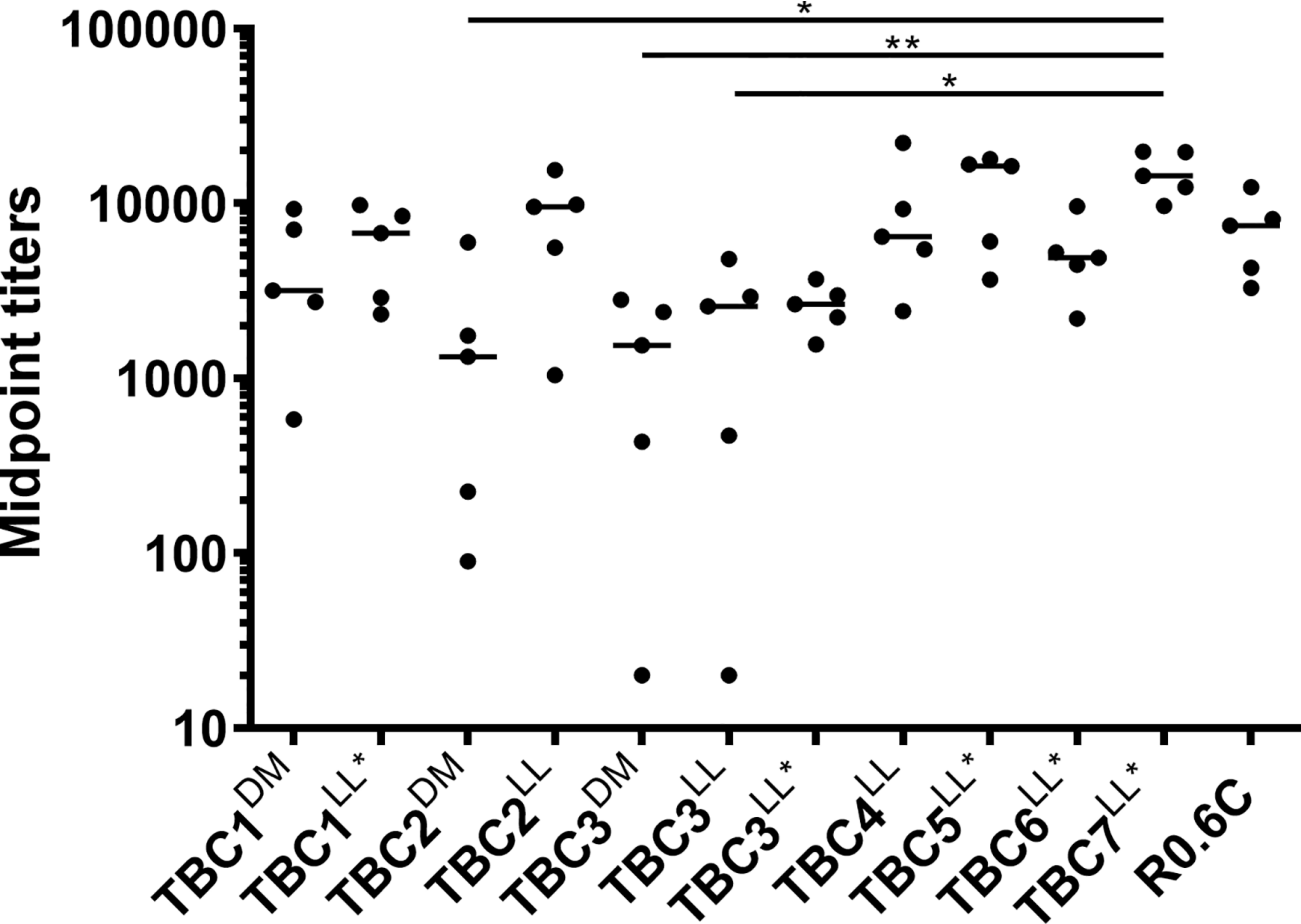
Immunogenicity of antigens in mice. Mice were immunized three times with 10 µg formulated in Montanide™ ISA720. Midpoint titers (EC50) values of individual mice sera (n=5) from the final bleed (day 56) analyzed in recombinant antigen ELISA against immunogen. Median values per group are shown as bars. Midpoint titers were calculated using sigmoidal curve fitting. For one mouse in TBC3^DM^ and one in TBC3^LL^ we could not fit a curve due to low levels of antibodies, and the midpoint titers were therefore arbitrarily set to 20. Names with * indicate antigens that were expressed as R0 chimeras. Differences between groups were tested for statistical significance the non-parametric Kruskal-Wallis test and Dunn’s multiple comparison post-test. Statistically significant differences are indicated in the figure (*<0.05, **<0.01), absence of symbols indicated no statistical significance.

We ultimately produced 11 protein fragments, covering 7 unique proteins, with sufficient yield for immunization studies (**Table 2**). We immunized 11 groups of five CD-1 mice with these recombinant antigens formulated in Montanide™ ISA720. Additionally, one positive control group was immunized with the Pfs48/45-based vaccine candidate R0.6C (31). Antibody titers from individual final bleed sera were analyzed in antigen ELISAs (**Figure 3**). All antigens induced antigen-specific antibodies, although some mice that were immunized with TBC2^DM^, TBC3^DM^ or TBC3^LL*^ had relatively low titers (Midpoint titers < 500).

**Table 2:** Recombinant proteins used in mouse immunization study.

Study ID	Protein	Expression system	Molecular Weight (kDa)
TBC1	1143700	DM	13.0
		LL*	72.3
TBC2	1306500 C	DM	16.3
		LL	16.7
TBC3	1348000	DM	14.7
		LL	14.8
		LL*	70.3
TBC4	0305300	LL	50.5
TBC5	R0.1014300	LL*	108.0
TBC6	R0.1314500	LL*	73.3
TBC7	R0.1433200B	LL*	94.9

DM, Drosophila melanogaster; LL, Lactococcus lactis; * indicate antigens that were expressed as R0 chimeras.

### Antibodies From Immunized Mice Recognize Native *P. falciparum* Antigens

**Figure 4:**
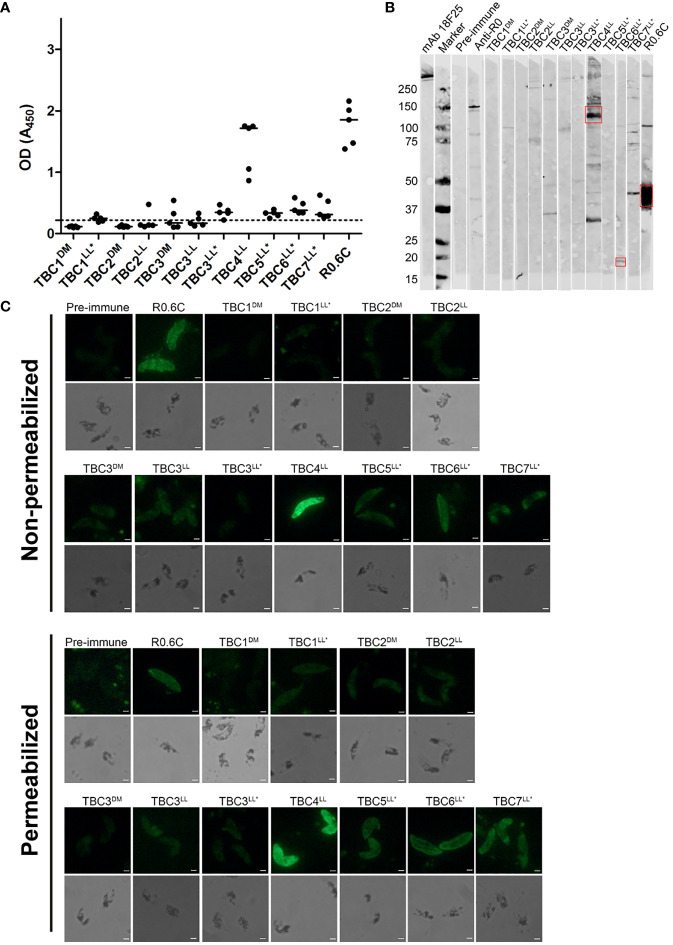
Reactivity of antibodies with native gametocyte antigens. Antibody reactivity to antigens in gametocyte extracts was assessed by **(A)** ELISA and **(B)** western blot. **(A)** Raw optical density (OD) values are presented for each individual mouse antisera with the median against native gametocyte extract ELISA. The dotted line represents the mean of all pre-immune sera plus three times the standard deviation. Samples with OD values above this cut-off are considered positive. **(B)** The sera that showed recognition were repeated in another western blot with comparable results (Data not shown). Red boxes indicate the putative protein of interest. **(C)** Reactivity with whole gametocytes was analyzed by immunofluorescence assays. Fixed gametocytes were incubated with 1:100 dilution of sera under non-permeabilizing and permeabilizing conditions. Scale bar represents 10 µm. Names with * indicate antigens that were expressed as R0 chimeras.

The reactivity of antibodies to native antigens present in gametocyte extract was assessed by ELISA and western blot (**Figure 4**). Clear reactivity in ELISA is observed for TBC4^LL^ and positive control group R0.6C (**Figure 4A**). All groups that were immunized with R0-fused antigens showed responses above the pre-immune values. However, since we had no group immunized with solely R0, we cannot determine whether these responses are specific to the TBC component or caused by antibodies against R0. A gametocyte western blot was performed in order to assess antigen-specificity of induced antibodies (**Figure 4B**). Antibodies induced by TBC4^LL^ and TBC6^LL*^ recognize protein at the expected sizes of 115kDa and 24.4kDa, respectively. TBC4^LL^-induced antibodies also react with a protein at a size around 30kDa; whether this is a processed form or whether the antibodies cross-react with other antigens is unclear. TBC7^LL*^ antibodies react with multiple proteins, but there is no clear reactivity with a product at the expected size of 152kDa. Furthermore, there is clear recognition of Pfs48/45 by R0.6C-induced antibodies.

Additionally, we performed immunofluorescence assays with whole gametocytes and gametes. Gametocytes were recognized by antibodies from TBC groups 4^LL^, 5^LL*^, 6^LL*^, 7^LL*^ and the R0.6C control group (**Figure 4C**). There is no clear difference between recognition of non-permeabilized and permeabilized cells, indicating that antibodies are primarily reacting with extracellular antigens. Interestingly, antibodies targeting TBC6^LL*^ seem not to react with stage V gametocytes, but appear to only recognize remaining immature gametocytes present in the culture (**Supplementary Figure 4**). Next, we analyzed the reactivity of antibodies with the surface of activated female gametes (**Supplementary Figure 5**). While the R0.6C positive control group showed clear recognition of gamete surfaces, the TBC1^DM^ group showed weak and patchy surface recognition and none of the other groups showed any reactivity.

### IgGs Induced by Novel TBV Candidates Do Not Reduce Transmission

**Figure 5:**
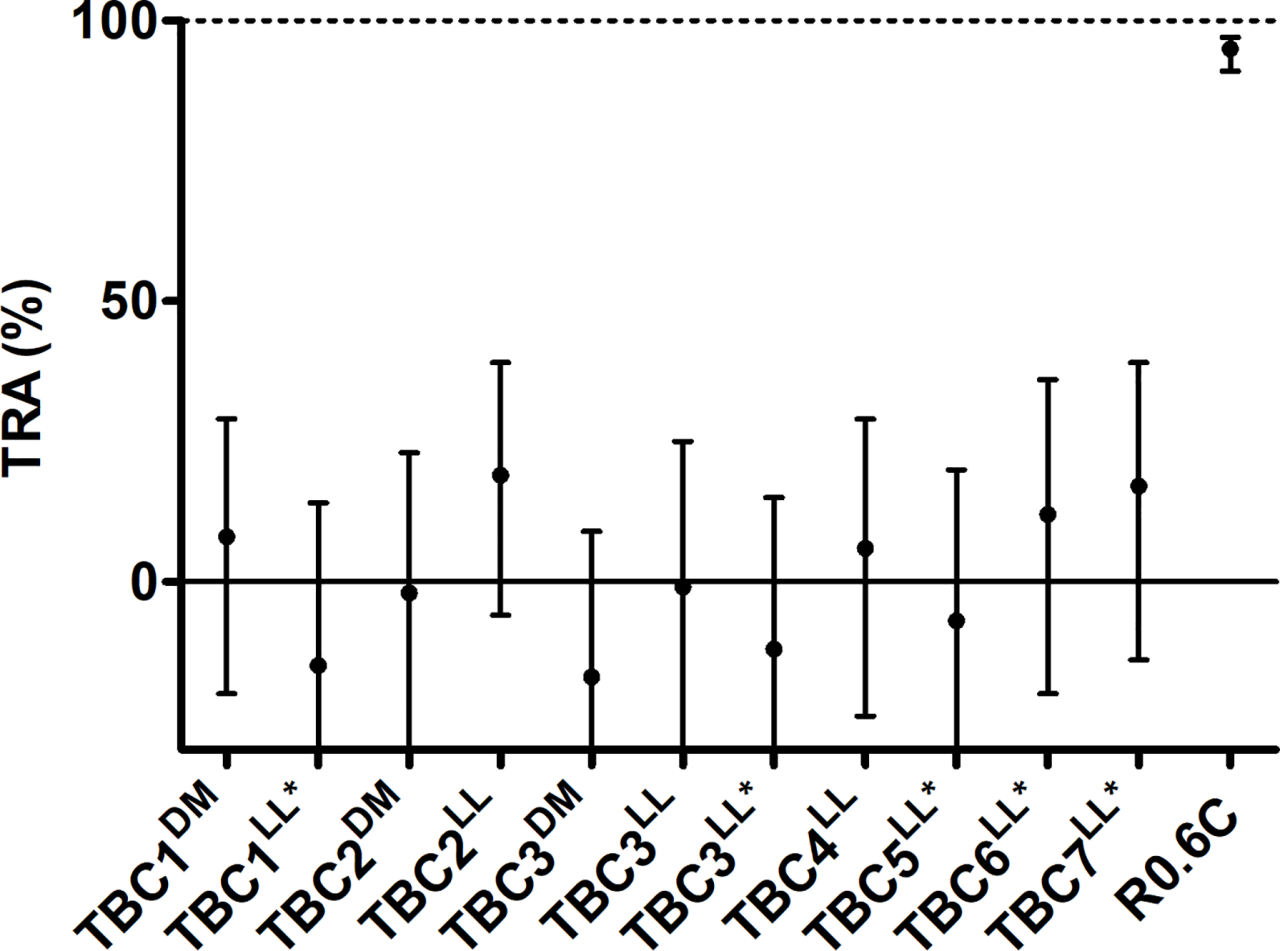
IgGs from mice immunized with novel transmission blocking vaccine candidates do not reduce transmission. Purified IgGs were tested in a single standard membrane feeding assay at a final concentration of 750 µg/mL except for TBC1^LL*^ and TBC3^LL*^, they were tested at 540 and 590 µg/mL due to low IgG yield, respectively. Transmission reducing activity (% TRA) estimates are shown with 95% confidence intervals. R0.6C showed statistical significant TRA (p<0.001), while none of the other groups showed significant TRA. Names with * indicate antigens that were expressed as R0 chimeras. Note that for TBC7^LL*^ only 17 mosquitoes had fed.

Total IgGs were purified from pooled sera of each group of immunized mice and were tested in the standard membrane feeding assay to assess functional TRA. Purified IgGs were mixed with cultured *P. falciparum* NF54 gametocytes and active human complement, and were fed to *A. stephensi* mosquitoes. None of the IgGs induced by the novel TBV candidates were able to reduce parasite transmission (range -15% till 22% TRA), while R0.6C-induced IgGs reduced transmission by 95% (**Figure 5**). Thus, while some of these sera react to native gametocyte antigens, they are unable to confer TRA in the model as tested here.

## Discussion

We successfully expressed 11 sexual stage *P. falciparum* antigens, covering seven proteins, in either *D. melanogaster* S2 cells or *L. lactis* to determine their potential as TBV candidates. We showed that these proteins were recognized by antibodies from naturally exposed individuals and induced antigen-specific antibody responses in mouse immunization studies. Mouse antibodies elicited by several antigens showed reactivity with native gametocyte proteins. None of the novel TBV candidates induced antibodies that reduced *P. falciparum* parasite development in the mosquito midgut as assessed in the standard membrane feeding assay.

Difficulties with the heterologous expression of *Plasmodium* proteins forms a major hurdle in the progress of TBV development. Expression of *Plasmodium* proteins is notoriously difficult, due to the A/T richness in the genome (19), high abundance of repeat regions (37) and the presence of disulphide bonds in extracellular proteins. This has hampered the high-throughput expression and evaluation of large antigen panels for the identification of novel TBV candidates.

In this study, we used both *D. melanogaster* S2 cells and *L. lactis* to express 12 putative surface expressed gametocyte antigens that have been associated with TRA (16). The advantage of eukaryotic expression systems is their ability to introduce post-translational modifications, including disulphide bond formation. Disulphide bond formation can be important for correct folding of extracellular proteins, as demonstrated for the TBV candidate R0.6C (38, 39). Our study is the first to use S2 cells for the expression of an extensive list of malarial antigens. We detected protein expression by S2 cells for 5 out of 18 antigens (28%). We experienced difficulties in obtaining highly pure protein preparations, because some high molecular weight host proteins also bind to the nickel affinity column. More antigens might have been recovered if a more specific antibody-based affinity purification was used, such as C-tag (EPEA) (23, 40).

We ultimately immunized mice with three antigens expressed in S2 cells. The TBC1^DM^ sequence contains a single cysteine, and the antigen appears to fully dimerize through the formation of a disulphide bond. Although we do not know whether dimerization prevented the induction of functional antibodies, one could consider mutation of the cysteine to prevent dimerization. A possible drawback of the use of a eukaryotic expression system for *Plasmodium* antigens is the glycosylation of expressed proteins, which could potentially mask important epitopes. It has been a matter of debate to what extent *Plasmodium* species are capable of glycosylating proteins. So far, only short and truncated *O*- and *N*-glycans have been identified in *Plasmodium* blood stage parasites (41). We showed that TBC2^DM^ was glycosylated by S2 cells, but whether the short glycans that are attached by S2 cells (42) impacted the induction of functional antibodies by TBC2 needs to be further investigated.

The use of *L. lactis* has the advantage that it is a low-cost, easily scalable and ‘generally recognized as safe’ (GRAS) system for protein expression. It has already proved to be a suitable system for the expression of complex *P. falciparum* antigens (24, 43, 44). Furthermore, this prokaryotic system does not introduce glycans, which may be beneficial. We were able to produce all the S2 cell expressed antigens also in *L. lactis*, but additionally produced four antigens exclusively in *L. lactis*. One of the antigens that was expressed in both expression systems, TBC2, showed noticeably higher recognition by endemic plasma samples when expressed in S2 cells, possibly due to differences in overall folding. To cover possible differences between expression systems, we decided to immunize with all successfully purified antigens including those produced in both systems.

Antibodies induced by Pf3D7_0305300 (TBC4^LL^) showed high reactivity with gametocytes in ELISA, western blot and IFA. The antigen appears to localize to the membrane gametocytes. The recognition of native protein by mouse antibodies together with the reactivity of antibodies in endemic plasma confirm the presence of native epitopes in the recombinant protein. Interestingly, antibody levels in endemic plasma samples with >90% TRA were significantly higher than in samples with <10% TRA, suggesting that these antibodies may indeed be associated with TRA. However, it should be noted that this association does not prove causality; affinity purification of antibodies against TBC4^LL^ could provide this evidence. Moreover, findings were based on a very limited set of plasma samples and larger sero-epidemiological studies are needed to confirm the association and the lack of association for several other antigens. Despite the strong recognition of gametocytes, antibodies elicited in mice were not able to reduce parasite transmission in the SMFA that is the standard assay to prioritize TBV candidates for further study (45). We observed no antibody recognition of proteins on the surface of female gametes, which might explain the apparent absence of functional immune responses in the SMFA. Currently, there is very limited information available regarding the biological role of this protein. InterPro predicts homology to a Major Facilitator Superfamily Transporter and a Histone acetyl transferase GCN5 as reported on PlasmoDB (30). Elucidating the expression and function of this protein could provide information whether naturally acquired antibodies to this protein are biomarkers of gametocyte exposure or have a mechanistic role in TRA.

Antibodies induced by Pf3D7_1314500 (TBC6^LL*^) showed specific reactivity by western blot and weak reactivity was also seen in gametocyte ELISA. Interestingly, in the gametocyte IFA only a subset of gametocytes was recognized (**Supplementary Figure 4**). The gametocytes that were recognized display an elongated shape with a pointed tip suggesting these are immature gametocytes (46). We used gametocyte cultures with a minority of remaining immature parasites to prepare gametocyte extracts for ELISA and western blot. Based on the lack of expression in stage V gametocytes this might explain the observed low-level reactivity in those assays. The protein has been classified as a putative transmembrane emp24 domain-containing protein (PlasmoDB). Transmembrane emp24 domain proteins (TMED) belong to a widely conserved protein family present in all eukaryotes (47). They are important regulators of protein transport between the endoplasmic reticulum and Golgi (48). Although the specific function of Pf3D7_1314500 needs be further investigated, this predicted function in protein transport makes it unlikely that this antigen is a promising TBV candidate. However, a recent study showed that increased antibody responses to Pf3D7_1314500 in two cohorts of malaria-exposed individuals were significantly associated with gametocytemia, demonstrating a potential role as biomarker of gametocyte carriage (49).

Overall, we observed limited antibody reactivity with sexual stage parasites and none of the mouse sera was able to reduce parasite transmission. Our study has several uncertainties that are important to consider. The selected TBV candidates in our study were solely based on TRA-associated antibody responses in the protein microarray study by Stone et al. (16). Although antibodies to the 12 TBV candidates were associated with TRA, it does not mean they all play a mechanistic role in TRA but may be biomarkers of gametocyte exposure. In addition, the antigens on the microarray were selected based on observed expression in stage V gametocytes in a single proteomic study (50). However, we do not know to what extent these proteins are expressed *in vivo* and whether they are present at detectable levels in the gametocyte lysate or whole sexual parasites used in our assays. Furthermore, in this study we only assessed reactivity with gametocytes and the surface of female gametes. In order to fully assess reactivity to sexual stages, future experiments should include male gametes. Additionally, we do not know whether the recombinantly produced antigen fragments are properly folded and presenting all native epitopes, which may be essential for the induction of functional antibodies. Lastly, since the majority of antigens were too large to express, we selected fragments based on the initial protein microarray data and taking into account predicted domain boundaries. It cannot be ruled out that this fragment selection has affected the recombinant expression and overall folding of antigens or the induction of functional antibodies. Transmission reducing epitopes may have been absent in the fragments or outcompeted by the presence of immunodominant epitopes. The latter has been observed for a Pfs47 vaccine construct, where the full-length protein was unable to induce functional antibodies, while a small fragment could elicit antibodies with TRA (13). Even though we were unable to detect any functional activity, our data may provide novel leads to improve our limited understanding of gametocyte biology. We showed that the putative transporter Pf3D7_0305300 is abundantly expressed in gametocytes, and it will be of great interest to further investigate the function of this protein.

In conclusion, we expressed 11 fragments covering seven sexual stage *P. falciparum* proteins in common heterologous expression systems and analyzed these in a mouse immunization study. We observed limited reactivity to native parasite proteins by antibodies from immunized mice and the induced antibodies were not able to reduce parasite transmission. Based on our data, it is difficult to conclude whether any of the antigens should be considered a viable TBV candidate. Future studies focusing on phenotypic analysis of these antigens by generating *P. falciparum* knock-out lines with additional fluorescent protein tagging could provide useful information on localization and potential biological function. This would help to further prioritize promising TBV candidates.

## Data Availability Statement

The original contributions presented in the study are included in the article/**Supplementary Material**. Further inquiries can be directed to the corresponding author.

## Ethics Statement

The animal study was reviewed and approved by Radboud University’s Animal Experiment Committee (DierExperimentenCommissie, RUDEC).

## Author Contributions

RJ, TB, and MJ conceived and designed the study. RJ, MJ, EL, and JP designed protein fragments for expression. SS and MT designed and supervised *L. lactis* expression experiments. RJ, KT, MV-B, and G-JG performed experiments. MJ supervised the project. RJ, TB, and MJ wrote the manuscript with input from all authors. All authors contributed to the article and approved the submitted version.

## Funding

This was work was supported by the European Union’s Horizon 2020 research and innovation program under grant agreement No. 733273 and by PATH’s Malaria Vaccine Initiative. TB is supported by a fellowship from the European Research Council (ERC-CoG 864180; QUANTUM). MJ is supported by the Netherlands Organization for Scientific Research (Vidi fellowship NWO project number 192.061). WS is supported by a Sir Henry Wellcome Fellowship from the Wellcome Trust (number 218676/Z/19/Z).

## Conflict of Interest

The authors declare that the research was conducted in the absence of any commercial or financial relationships that could be construed as a potential conflict of interest.

## Publisher’s Note

All claims expressed in this article are solely those of the authors and do not necessarily represent those of their affiliated organizations, or those of the publisher, the editors and the reviewers. Any product that may be evaluated in this article, or claim that may be made by its manufacturer, is not guaranteed or endorsed by the publisher.

## References

[1] Graumans W Jacobs E Bousema T Sinnis P. When Is a Plasmodium-Infected Mosquito an Infectious Mosquito? Trends Parasitol (2020) 36(8):705–16. doi: 10.1016/j.pt.2020.05.011 PMC7386819 32620501

[2] Cibulskis RE Alonso P Aponte J Aregawi M Barrette A Bergeron L. Malaria: Global Progress 2000 - 2015 and Future Challenges. Infect Dis Poverty (2016) 5(1):61. doi: 10.1186/s40249-016-0151-8 27282148 PMC4901420

[3] Sinnis P Coppi A. A Long and Winding Road: The Plasmodium Sporozoite's Journey in the Mammalian Host. Parasitol Int (2007) 56(3):171–8. doi: 10.1016/j.parint.2007.04.002 PMC1995443 17513164

[4] Vermeulen AN Ponnudurai T Beckers PJ Verhave JP Smits MA Meuwissen JH. Sequential Expression of Antigens on Sexual Stages of *Plasmodium falciparum* Accessible to Transmission-Blocking Antibodies in the Mosquito. J Exp Med (1985) 162(5):1460–76. doi: 10.1084/jem.162.5.1460 PMC2187939 2865324

[5] Talaat KR Ellis RD Hurd J Hentrich A Gabriel E Hynes NA. Safety and Immunogenicity of Pfs25-EPA/Alhydrogel(R), A Transmission Blocking Vaccine Against *Plasmodium falciparum*: An Open Label Study in Malaria Naive Adults. PloS One (2016) 11(10):e0163144. doi: 10.1371/journal.pone.0163144 27749907 PMC5066979

[6] Sagara I Healy SA Assadou MH Gabriel EE Kone M Sissoko K. Safety and Immunogenicity of Pfs25H-EPA/Alhydrogel, a Transmission-Blocking Vaccine Against Plasmodium Falciparum: A Randomised, Double-Blind, Comparator-Controlled, Dose-Escalation Study in Healthy Malian Adults. Lancet Infect Dis (2018) 18(9):969–82. doi: 10.1016/S1473-3099(18)30344-X PMC6287938 30061051

[7] Healy SA Anderson C Swihart BJ Mwakingwe A Gabriel EE Decederfelt H. Pfs230 Yields Higher Malaria Transmission-Blocking Vaccine Activity Than Pfs25 in Humans But Not Mice. J Clin Invest (2021) 131(7):e146221. doi: 10.1172/JCI146221 PMC8011888 33561016

[8] Liu Y Tewari R Ning J Blagborough AM Garbom S Pei J. The Conserved Plant Sterility Gene HAP2 Functions After Attachment of Fusogenic Membranes in Chlamydomonas and Plasmodium Gametes. Genes Dev (2008) 22(8):1051–68. doi: 10.1101/gad.1656508 PMC2335326 18367645

[9] van Schaijk BC van Dijk MR van de Vegte-Bolmer M van Gemert GJ van Dooren MW Eksi S. Pfs47, Paralog of the Male Fertility Factor Pfs48/45, Is a Female Specific Surface Protein in *Plasmodium falciparum* . Mol Biochem Parasitol (2006) 149(2):216–22. doi: 10.1016/j.molbiopara.2006.05.015 16824624

[10] Blagborough AM Sinden RE. *Plasmodium berghei* HAP2 Induces Strong Malaria Transmission-Blocking Immunity *In Vivo* and *In Vitro* . Vaccine (2009) 27(38):5187–94. doi: 10.1016/j.vaccine.2009.06.069 19596419

[11] Miura K Takashima E Deng B Tullo G Diouf A Moretz SE. Functional Comparison of *Plasmodium falciparum* Transmission-Blocking Vaccine Candidates by the Standard Membrane-Feeding Assay. Infect Immun (2013) 81(12):4377–82. doi: 10.1128/IAI.01056-13 PMC3838000 24042109

[12] Angrisano F Sala KA Da DF Liu Y Pei J Grishin NV. Targeting the Conserved Fusion Loop of HAP2 Inhibits the Transmission of Plasmodium Berghei and Falciparum. Cell Rep (2017) 21(10):2868–78. doi: 10.1016/j.celrep.2017.11.024 PMC5732318 29212032

[13] Canepa GE Molina-Cruz A Yenkoidiok-Douti L Calvo E Williams AE Burkhardt M. Antibody Targeting of a Specific Region of Pfs47 Blocks *Plasmodium falciparum* Malaria Transmission. NPJ Vaccines (2018) 3:26. doi: 10.1038/s41541-018-0065-5 30002917 PMC6039440

[14] Molina-Cruz A Canepa GE Barillas-Mury C. Plasmodium P47: A Key Gene for Malaria Transmission by Mosquito Vectors. Curr Opin Microbiol (2017) 40:168–74. doi: 10.1016/j.mib.2017.11.029 PMC5739336 29229188

[15] Yenkoidiok-Douti L Williams AE Canepa GE Molina-Cruz A Barillas-Mury C. Engineering a Virus-Like Particle as an Antigenic Platform for a Pfs47-Targeted Malaria Transmission-Blocking Vaccine. Sci Rep (2019) 9(1):16833. doi: 10.1038/s41598-019-53208-z 31727945 PMC6856133

[16] Stone WJR Campo JJ Ouedraogo AL Meerstein-Kessel L Morlais I Da D. Unravelling the Immune Signature of Plasmodium Falciparum Transmission-Reducing Immunity. Nat Commun (2018) 9(1):558. doi: 10.1038/s41467-017-02646-2 29422648 PMC5805765

[17] Nikolaeva D Illingworth JJ Miura K Alanine DGW Brian IJ Li Y. Functional Characterization and Comparison of *Plasmodium falciparum* Proteins as Targets of Transmission-Blocking Antibodies. Mol Cell Proteomics (2020) 19(1):155–66. doi: 10.1074/mcp.RA117.000036 PMC6944241 29089373

[18] Tripathi AK Oakley MS Verma N Mlambo G Zheng H Meredith SM. Plasmodium Falciparum Pf77 and Male Development Gene 1 as Vaccine Antigens That Induce Potent Transmission-Reducing Antibodies. Sci Transl Med (2021) 13(597):eabg2112. doi: 10.1126/scitranslmed.abg2112 34108248 PMC11018285

[19] Gardner MJ Hall N Fung E White O Berriman M Hyman RW. Genome Sequence of the Human Malaria Parasite Plasmodium Falciparum. Nature (2002) 419(6906):498–511. doi: 10.1038/nature01097 12368864 PMC3836256

[20] Lasonder E Ishihama Y Andersen JS Vermunt AM Pain A Sauerwein RW. Analysis of the *Plasmodium falciparum* Proteome by High-Accuracy Mass Spectrometry. Nature (2002) 419(6906):537–42. doi: 10.1038/nature01111 12368870

[21] Khan SM Franke-Fayard B Mair GR Lasonder E Janse CJ Mann M. Proteome Analysis of Separated Male and Female Gametocytes Reveals Novel Sex-Specific Plasmodium Biology. Cell (2005) 121(5):675–87. doi: 10.1016/j.cell.2005.03.027 15935755

[22] de Jong RM Tebeje SK Meerstein-Kessel L Tadesse FG Jore MM Stone W. Immunity Against Sexual Stage *Plasmodium falciparum* and Plasmodium Vivax Parasites. Immunol Rev (2020) 293(1):190–215. doi: 10.1111/imr.12828 31840844 PMC6973022

[23] Lennartz F Brod F Dabbs R Miura K Mekhaiel D Marini A. Structural Basis for Recognition of the Malaria Vaccine Candidate Pfs48/45 by a Transmission Blocking Antibody. Nat Commun (2018) 9(1):3822. doi: 10.1038/s41467-018-06340-9 30237518 PMC6148045

[24] Singh SK Plieskatt J Chourasia BK Fabra-Garcia A Garcia-Senosiain A Singh V. A Reproducible and Scalable Process for Manufacturing a Pfs48/45 Based *Plasmodium falciparum* Transmission-Blocking Vaccine. Front Immunol (2020) 11:606266. doi: 10.3389/fimmu.2020.606266 33505395 PMC7832176

[25] Feng Z Hoffmann RN Nussenzweig RS Tsuji M Fujioka H Aikawa M. Pfs2400 can Mediate Antibody-Dependent Malaria Transmission Inhibition and may be the *Plasmodium falciparum* 11.1 gene product. J Exp Med (1993) 177(2):273–81. doi: 10.1084/jem.177.2.273 PMC2190917 8426106

[26] Nielsen H Tsirigos KD Brunak S Von Heijne G. A Brief History of Protein Sorting Prediction. Protein J (2019) 38(3):200–16. doi: 10.1007/s10930-019-09838-3 PMC6589146 31119599

[27] Krogh A Larsson B von Heijne G Sonnhammer EL. Predicting Transmembrane Protein Topology With a Hidden Markov Model: Application to Complete Genomes. J Mol Biol (2001) 305(3):567–80. doi: 10.1006/jmbi.2000.4315 11152613

[28] Sonnhammer EL von Heijne G Krogh A. A Hidden Markov Model for Predicting Transmembrane Helices in Protein Sequences. Proc Int Conf Intell Syst Mol Biol (1998) 6:175–82. 9783223

[29] Drozdetskiy A Cole C Procter J Barton GJ. JPred4: A Protein Secondary Structure Prediction Server. Nucleic Acids Res (2015) 43(W1):W389–94. doi: 10.1093/nar/gkv332 PMC4489285 25883141

[30] Aurrecoechea C Brestelli J Brunk BP Dommer J Fischer S Gajria B. PlasmoDB: A Functional Genomic Database for Malaria Parasites. Nucleic Acids Res (2009) 37(Database issue):D539–43. doi: 10.1093/nar/gkn814 PMC2686598 18957442

[31] Singh SK Roeffen W Mistarz UH Chourasia BK Yang F Rand KD. Construct Design, Production, and Characterization of *Plasmodium falciparum* 48/45 R0.6C Subunit Protein Produced in Lactococcus Lactis as Candidate Vaccine. Microb Cell Fact (2017) 16(1):97. doi: 10.1186/s12934-017-0710-0 28569168 PMC5452637

[32] Singh SK Roeffen W Andersen G Bousema T Christiansen M Sauerwein R. A *Plasmodium falciparum* 48/45 Single Epitope R0.6C Subunit Protein Elicits High Levels of Transmission Blocking Antibodies. Vaccine (2015) 33(16):1981–6. doi: 10.1016/j.vaccine.2015.02.040 25728318

[33] Holo H Nes IF. Transformation of Lactococcus by Electroporation. Methods Mol Biol (1995) 47:195–9. doi: 10.1385/0-89603-310-4:195 7550735

[34] Theisen M Roeffen W Singh SK Andersen G Amoah L van de Vegte-Bolmer M. A Multi-Stage Malaria Vaccine Candidate Targeting Both Transmission and Asexual Parasite Life-Cycle Stages. Vaccine (2014) 32(22):2623–30. doi: 10.1016/j.vaccine.2014.03.020 24662702

[35] Stone WJ Eldering M van Gemert GJ Lanke KH Grignard L van de Vegte-Bolmer MG. The Relevance and Applicability of Oocyst Prevalence as a Read-Out for Mosquito Feeding Assays. Sci Rep (2013) 3:3418. doi: 10.1038/srep03418 24301557 PMC4894383

[36] Amoah LE Nuvor SV Obboh EK Acquah FK Asare K Singh SK. Natural Antibody Responses to *Plasmodium falciparum* MSP3 and GLURP(R0) Antigens Are Associated With Low Parasite Densities in Malaria Patients Living in the Central Region of Ghana. Parasit Vectors (2017) 10(1):395. doi: 10.1186/s13071-017-2338-7 28835262 PMC5569498

[37] Davies HM Nofal SD McLaughlin EJ Osborne AR. Repetitive Sequences in Malaria Parasite Proteins. FEMS Microbiol Rev (2017) 41(6):923–40. doi: 10.1093/femsre/fux046 PMC5812512 29077880

[38] Theisen M Jore MM Sauerwein R. Towards Clinical Development of a Pfs48/45-Based Transmission Blocking Malaria Vaccine. Expert Rev Vaccines (2017) 16(4):329–36. doi: 10.1080/14760584.2017.1276833 28043178

[39] Outchkourov N Vermunt A Jansen J Kaan A Roeffen W Teelen K. Epitope Analysis of the Malaria Surface Antigen Pfs48/45 Identifies a Subdomain That Elicits Transmission Blocking Antibodies. J Biol Chem (2007) 282(23):17148–56. doi: 10.1074/jbc.M700948200 17426022

[40] Jin J Hjerrild KA Silk SE Brown RE Labbe GM Marshall JM. Accelerating the Clinical Development of Protein-Based Vaccines for Malaria by Efficient Purification Using a Four Amino Acid C-Terminal 'C-Tag'. Int J Parasitol (2017) 47(7):435–46. doi: 10.1016/j.ijpara.2016.12.001 PMC5482323 28153778

[41] Goerdeler F Seeberger PH Moscovitz O. Unveiling the Sugary Secrets of Plasmodium Parasites. Front Microbiol (2021) 12:712538. doi: 10.3389/fmicb.2021.712538 34335547 PMC8322443

[42] de Jongh WA Sancha,S Dyring C. The Use of Drosophila S2 Cells in R&D and Bioprocessing. Pharm Bioprocess (2013) 1(2):197–213. doi: 10.4155/pbp.13.18

[43] Singh SK Tiendrebeogo RW Chourasia BK Kana IH Singh S Theisen M. Lactococcus Lactis Provides an Efficient Platform for Production of Disulfide-Rich Recombinant Proteins From *Plasmodium falciparum* . Microb Cell Fact (2018) 17(1):55. doi: 10.1186/s12934-018-0902-2 29618355 PMC5885415

[44] Singh SK Plieskatt J Chourasia BK Singh V Bolscher JM Dechering KJ. The *Plasmodium falciparum* Circumsporozoite Protein Produced in Lactococcus Lactis Is Pure and Stable. J Biol Chem (2020) 295(2):403–14. doi: 10.1074/jbc.RA119.011268 PMC6956534 31792057

[45] Churcher TS Blagborough AM Delves M Ramakrishnan C Kapulu MC Williams AR. Measuring the Blockade of Malaria Transmission–an Analysis of the Standard Membrane Feeding Assay. Int J Parasitol (2012) 42(11):1037–44. doi: 10.1016/j.ijpara.2012.09.002 23023048

[46] Carter R Miller LH. Evidence for Environmental Modulation of Gametocytogenesis in *Plasmodium falciparum* in Continuous Culture. Bull World Health Organ (1979) 57 Suppl 1:37–52. 397008 PMC2395706

[47] Carney GE Bowen NJ. P24 Proteins, Intracellular Trafficking, and Behavior: Drosophila Melanogaster Provides Insights and Opportunities. Biol Cell (2004) 96(4):271–8. doi: 10.1111/j.1768-322X.2004.tb01415.x 15145531

[48] Aber R Chan W Mugisha S Jerome-Majewska LA. Transmembrane Emp24 Domain Proteins in Development and Disease. Genet Res (Camb) (2019) 101:e14. doi: 10.1017/S0016672319000090 31878985 PMC7045115

[49] Muthui MK Takashima E Omondi BR Kinya C Muasya WI Nagaoka H. Characterization of Naturally Acquired Immunity to a Panel of Antigens Expressed in Mature *P. falciparum* Gametocytes. Front Cell Infect Microbiol (2021) 11:774537. doi: 10.3389/fcimb.2021.774537 34869075 PMC8633105

[50] Lasonder E Rijpma SR van Schaijk BC Hoeijmakers WA Kensche PR Gresnigt MS. Integrated Transcriptomic and Proteomic Analyses of *P. falciparum* Gametocytes: Molecular Insight Into Sex-Specific Processes and Translational Repression. Nucleic Acids Res (2016) 44(13):6087–101. doi: 10.1093/nar/gkw536 PMC5291273 27298255

