# Supplementary material for: Heterologous Expression and Evaluation of Novel Plasmodium falciparum Transmission Blocking Vaccine Candidates
Source: Front Immunol. 2022 Jun 23;13:909060. doi: 10.3389/fimmu.2022.909060 (PMC9259988; doi:10.3389/fimmu.2022.909060)
Supplement: Supplementary file 1 [file DataSheet_1.docx]

**Supplementary files**

**
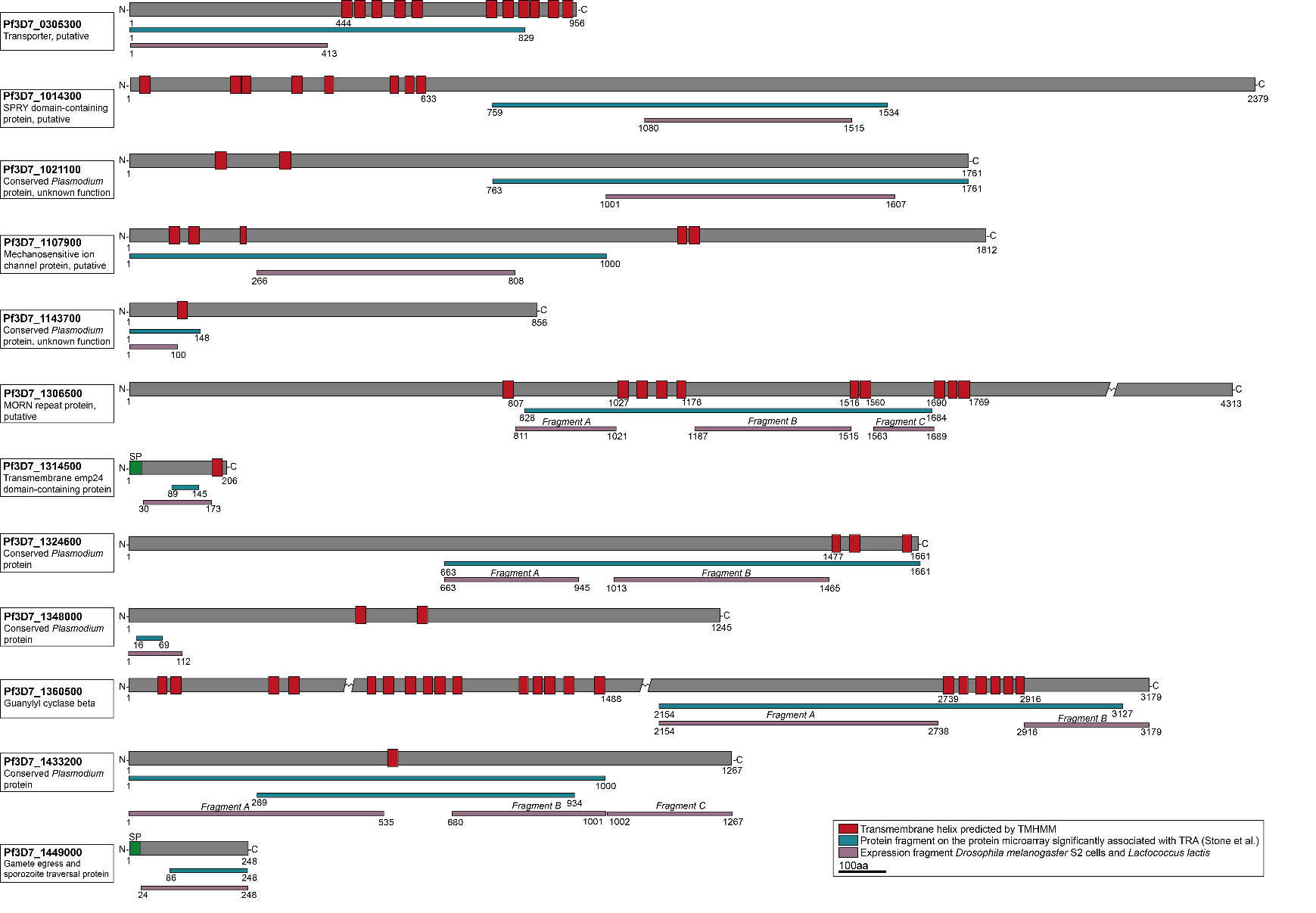
**

**Supplementary Figure 1:** Schematic overview of the proteins analyzed in this study, with in red the transmembrane domains and in green the signal peptides (SP) as predicted by TMHMM^27,28^ and SignalP^26^, respectively. The fragments that were printed on the protein microarray and to which antibody responses were significantly associated with transmission reducing activity (TRA) are shown in teal (Stone et al. 2018, Nat. Commun.). The fragments that were selected for expression in D*rosophila melanogaster S2 cells* and *Lactococcus lactis* are shown in purple*.* Amino acid numbers are depicted for fragment and protein boundaries. Due to the large size of 1306500 and 1365000, some of their parts are not shown.


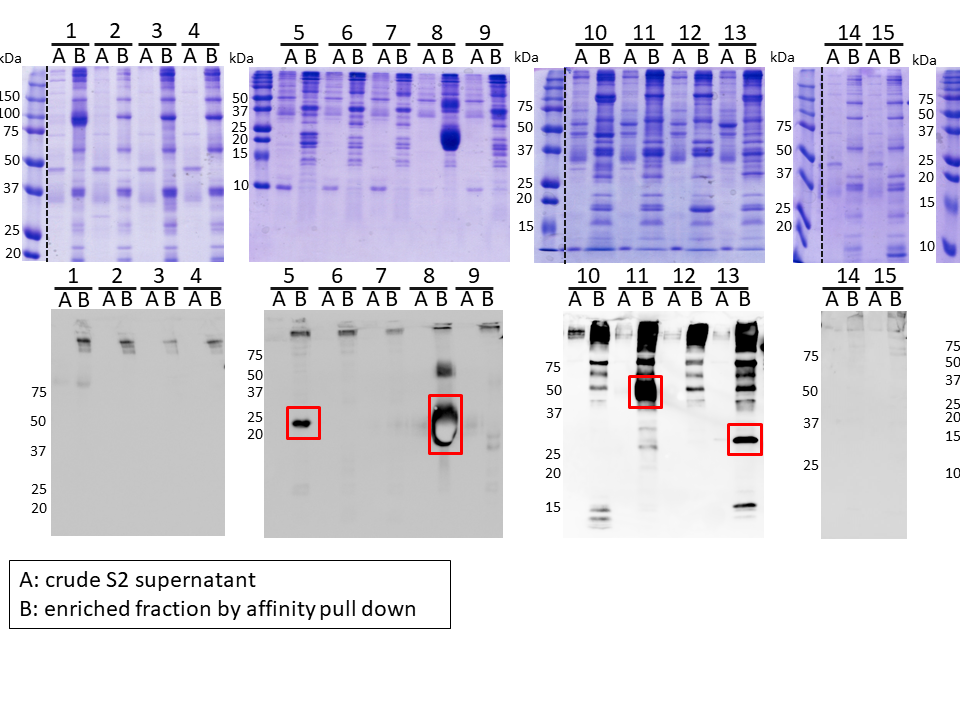

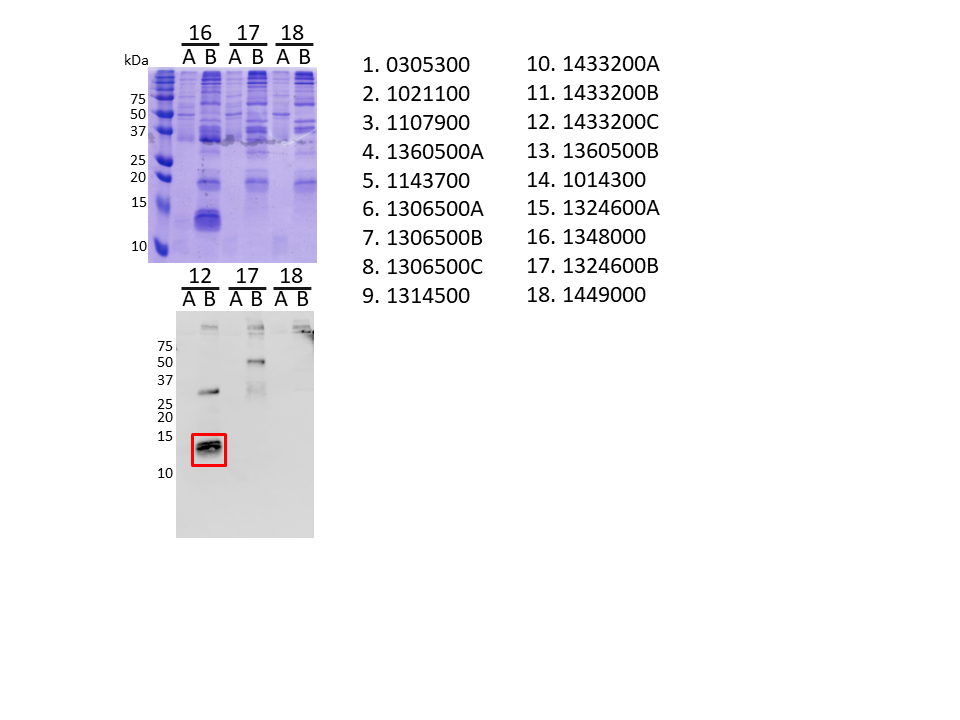


**Supplementary Figure 2:** Expression analysis of supernatant from stably transfected *Drosophila melanogaster* S2 cells by Coomassie-staining of non-reducing polyacrylamide gels and non-reducing western blot detection using an Anti-His antibody. Five antigens showed clear expression (red boxes) on western blot of expected monomeric protein. **
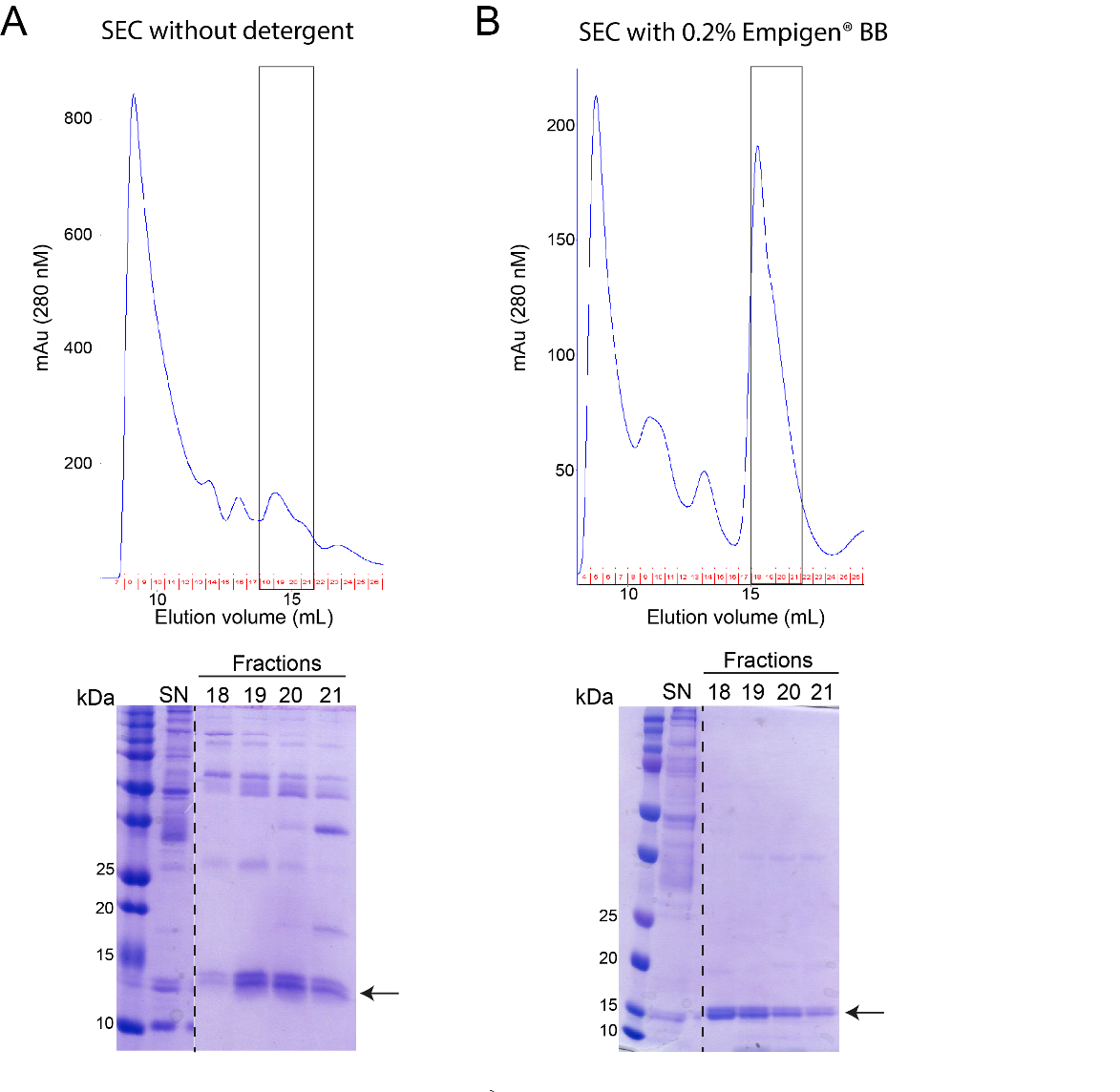
**

**Supplementary Figure 3:** A representative example of the purification of Pf3D7_1348000 (TBC3^DM^) from *Drosophila melanogaster* S2 cells supernatant without (**A**) and with (**B**) 0.2% Empigen® BB added during the purification process. The upper panel shows the chromatogram of the nickel purified sample ran on a Superdex 200 10/300 GL column and below a Coomassie-stained polyacrylamide gel loaded with the appropriate fractions. The arrow indicated the protein of interest. SN: Supernatant

**
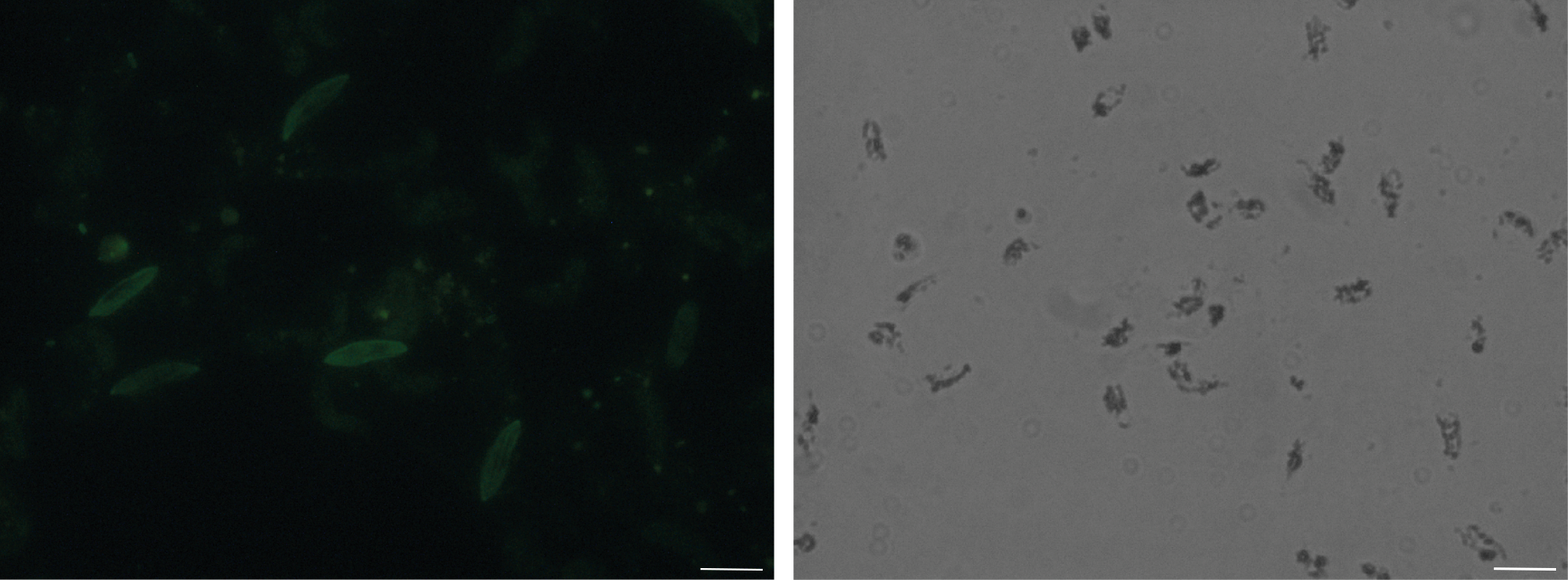
**

**Supplementary Figure 4:** Fixed and permeabilized gametocytes probed with sera from mice immunized with Pf3D7_1314500 (TBC6^LL*^). The black pigment indicates gametocytes on the brightfield image (right). Reactivity of sera is depicted in the fluorescent image (left). Only a subset of gametocytes is recognized by the sera. Scale bars represent 50µm.

**
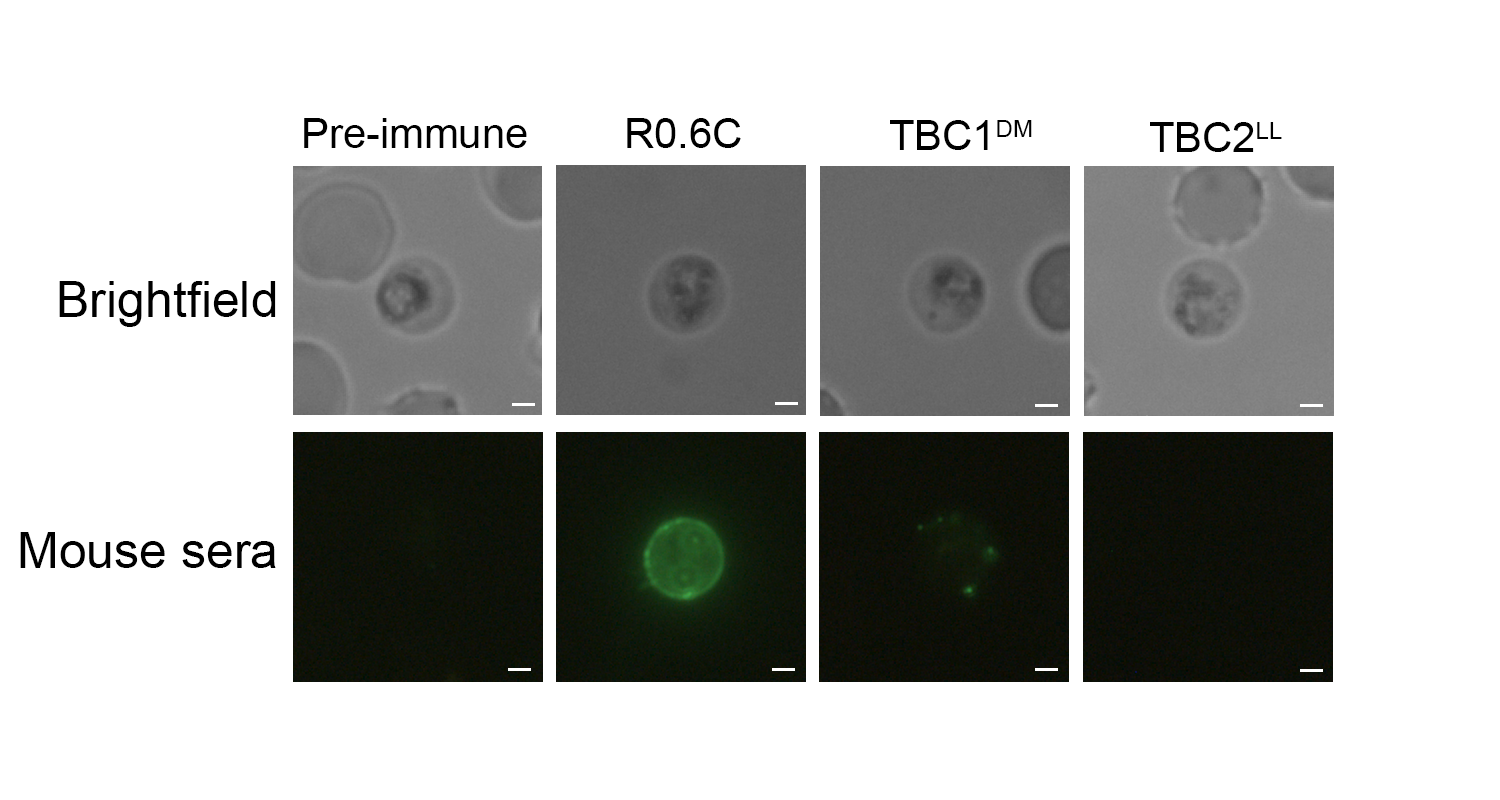
**

**Supplementary Figure 5:** Immunofluorescence assay of live female gametes with pooled final bleed mice serum. Sera were used at a 1:20 dilution. Scale bar represents 10µm.

**Supplementary Table 1:** primer sequences used to generate amplicons for expression in *Lactococcus lactis.*

| **Gene ID** |  | **Primer sequence** |
| --- | --- | --- |
| Pf3D7_0305300 | Forward | ggttcAGATCTATGGATGTTTATAAAGAAGAGAACTCC |
|  | Reverse | gggccGTCGACTTAGTGGTGATGGTGATGATGTCCTGAATATTTTTTTAAAACATCATCATTAAATAATG |
| Pf3D7_1014300 | Forward | ggttcAGATCTGATGAGGATAGCACACATAAAGATAG |
|  | Reverse | gggccGTCGACTTAGTGGTGATGGTGATGATGTCCTGATTTTTTATTATCTTCTGGTGTAACATC |
| Pf3D7_1021100 | Forward | ggttcAGATCTGATTTTATGGGATTTAGTAAAATATCAAC |
|  | Reverse | gggccGTCGACTTAGTGGTGATGGTGATGATGTCCTGAATGGGGTGAATTGAAAGTTAAAAAAGTA |
| Pf3D7_1107900 | Forward | ggttcAGATCTATGAATGCTCATGATTTACTTGGGAA |
|  | Reverse | gggccGTCGACTTAGTGGTGATGGTGATGATGTCCTGAATTATTATTATCATCATCATTATTATTATC |
| Pf3D7_1143700 | Forward | ggttcAGATCTAAAGTAAAAATAGGAAGCTTCGAAAATTT |
|  | Reverse | gggccGTCGACTTAGTGGTGATGGTGATGATGTCCTGAATCTTTTAATTTTACATTTATAAAATATGGA |
| Pf3D7_1306500 Fragment A | Forward | ggttcAGATCTATGCAAAGCCTTTCTTTTATATCCCA |
|  | Reverse – Nested | cgacaCAAATATGAATAATGTAAAATTTTGAATAG |
|  | Reverse | gggccGTCGACTTAGTGGTGATGGTGATGATGTCCTGATTCGTTTTCTTTTTTTTTTCTTTTTTTTTTTT |
| Pf3D7_1306500 Fragment B | Forward | ggttcAGATCTGAAAGTCAAGATAATCAACAGTTTCC |
|  | Reverse | gggccGTCGACTTAGTGGTGATGGTGATGATGTCCTGAAGTATACATATTTTCATTAAAAAGATTATG |
| Pf3D7_1306500 Fragment C | Forward | ggttcAGATCTGAACATCTATTTAAGAAATGTGATATAAG |
|  | Reverse | gggccGTCGACTTAGTGGTGATGGTGATGATGTCCTGATTTATTTTGTTTTTTACTATTATATAATTTTTTG |
| Pf3D7_1314500 | Forward | ggttcAGATCTGGACCATATGAAAAAGATTGTATTCAA |
|  | Reverse | gggccGTCGACTTAGTGGTGATGGTGATGATGTCCTGATTTGGAATTCATTTTTTCATTAAATTGTTT |
| Pf3D7_1324600 Fragment A | Forward | ggttcAGATCTGATGAAGGGGAGAATAAAACATTTG |
|  | Reverse | gggccGTCGACTTAGTGGTGATGGTGATGATGTCCTGAATTATCATTATTATTTATTACCATACACC |
| Pf3D7_1324600 Fragment B | Forward | ggttcAGATCTCATAGTGATTATATGAAAAGCATACAAA |
|  | Reverse | gggccGTCGACTTAGTGGTGATGGTGATGATGTCCTGAATCGTTTTGAATTTTTTCATTCTTTTCTA |
| Pf3D7_1348000 | Forward | ggttcGGATCCAGTTTAAATGGTGTAGATCTACATAC |
|  | Reverse | gggccGTCGACTTAGTGGTGATGGTGATGATGTCCTGACTTATACCCATCTATTTCTCTTTTTTG |
| Pf3D7_1360500 Fragment A | Forward | ggttcAGATCTTCTAAAGATGGTGTTTCTTATAATTTCC |
|  | Reverse | gggccGTCGACTTAGTGGTGATGGTGATGATGTCCTGATTTTTTTGTTAAATCAATATTAATGATTTC |
| Pf3D7_1360500 Fragment B | Forward | ggttcAGATCTTCAGAATATTTAGATAGAATACAATTCT |
|  | Reverse | gggccGTCGACTTAGTGGTGATGGTGATGATGTCCTGATGAACGTAATCCCTTTTTTTGACGTA |
| Pf3D7_1433200 Fragment A | Forward | ggttcAGATCTAATGAAAAGAAAAAACTTGATATTATCCG |
|  | Reverse | gggccGTCGACTTAGTGGTGATGGTGATGATGTCCTGATTCATATTTTTCTAGACCTATTAAAATATC |
| Pf3D7_1433200 Fragment B | Forward | ggttcGGATCCACATCTGATATCTCTTCTACCACAAC |
|  | Reverse | gggccGTCGACTTAGTGGTGATGGTGATGATGTCCTGAATTTAAATATTCTTGATTCTTTACATAATTTT |
| Pf3D7_1433200 Fragment C | Forwad | ggttcAGATCTGATGAAAACGGTATACATGATTATAAAG |
|  | Reverse - Nested | cgacaGTCTTACCTTTATATTTTAATAATACCAG |
|  | Reverse | gggccGTCGACTTAGTGGTGATGGTGATGATGTCCTGAATTTTTTTTTTTTTTTTTTTTCGGTAAGATA |
| Pf3D7_1449000 | Forward | ggttcAGATCTCATTCTGTAAATTATGCTCCCTTGAA |
|  | Reverse | gggccGTCGACTTAGTGGTGATGGTGATGATGTCCTGATTGTAATAGGGCTTTTTCTTGCTCTT |

Forward primer: overhang - BglII/BamHI site - annealing sequence

Reverse primer: overhang - SalI- stopcodon - His-tag - linker - annealing sequence

**Supplementary Table 2:** amino acid sequences of recombinantly expressed proteins.

| ***Drosophila melanogaster S2 cells*** | |
| --- | --- |
| 1143700 | **MKLCILLAVVAFVGLSLG***HHHHHH*AGMKVKIGSFENFLERLNNIKEEDIIINDENTEEMFSSFLITFYKENEGRYTIQEQEYINNLLNILINKIRDNKNGRNFFSNLLCIHFKSITPYFINVKLKD- |
| 1306500C | **MKLCILLAVVAFVGLSLG***HHHHHH*AGEHLFKKCDIRKEDLLTINDQHQNDNQFIDTYKTKEQIDHSIKTSINQKMNNHSTQTEIYQKENKYGKREKQKKNDENNKYNNDFENISNKQHNNVQTSTSSYQQSDIIEDNKNSRNKKLYNSKKQNK- |
| 1348000 | **MKLCILLAVVAFVGLSLG***HHHHHH*AGMSLNGVDLHTTDFINEKEEWMKKYENIELEYGALKKKFEEFSIDYKNKNEEIKNFATIKKDMILRYAYMKNENEFLKTQLNILILEKEEEEIKKRELIEINEKQKREIDGYK- |
| ***Lactococcus lactis*** | |
| 0305300 | **MKFNKKRVAIATFIALIFVSFFTISSIQDAQAAE**RSMDVYKEENSDLLNHQEEEISRNSNINDEFSETQLRDNVYEERENEYINKHNMMDDIQIMVDEQDNNNNYDNNNYYDDNINNYDNNNYDDNNYDDNNYDNNNNNSYYEYNQNAYEDNEPYNDYNDNNNNIINGSKYINEIQHNDKELYDMSYENNLNNEVLNVENRKKKSNNYDNVVNSNMGNNKNNTTANNNNNNHNNNNNNHSNNHSNNHSISHSNNHSNNHNNNHSNNHNKSHDVKDIPYCSFNNFQENEDKDGDKPGDVKSNNNMLRKKNSEENNAYNLFNNEKLKKLEKKKKKMLEKNKKKKKEDTLDDKKFSEVYNSIYENLKKKKEGKYNNDDNKDMMMMMMDDHKSNDNKHHKNNKHNNKHYNNNNDDDDDNDFYEINYNSSDNMNSVSSVEEDTLFNDDVLKKYSG*HHHHHH*- |
| 1306500C | **MKFNKKRVAIATFIALIFVSFFTISSIQDAQAA**ERSEHLFKKCDIRKEDLLTINDQHQNDNQFIDTYKTKEQIDHSIKTSINQKMNNHSTQTEIYQKENKYGKREKQKKNDENNKYNNDFENISNKQHNNVQTSTSSYQQSDIIEDNKNSRNKKLYNSKKQNKSG*HHHHHH*- |
| 1348000 | **MKFNKKRVAIATFIALIFVSFFTISSIQDAQAAE**GSSLNGVDLHTTDFINEKEEWMKKYENIELEYGALKKKFEEFSIDYKNKNEEIKNFATIKKDMILRYAYMKNENEFLKTQLNILILEKEEEEIKKRELIEINEKQKREIDGYKSG*HHHHHH*- |
| R0.1014300 | **MKFNKKRVAIATFIALIFVSFFTISSIQDAQAAE**RSTSENRNKRIGGPKLRGNVTSNIKFPSDNKGKIIRGSNDKLNKNSEDVLEQSEKSLVSENVPSGLDIDDIPKESIFIQEDQEGQTHSELNPETSEHSKDLNNNGSKNESSDIISENNKSNKVQNHFESLSDLELLENSSQDNLDKDTISTEPFPNQKHKDLQQDLNDEPLEPFPTQIHKDYKEKNLINEEDSEPFPRQKHKKVDNHNEEKNVFHENGSANGNQGSLKLKSFDEHLKDEKIENEPLVHENLSIPNDPIEQILNQPEQETNIQEQLYNEKQNVEEKQNSQIPSLDLKEPTNEDILPNHNPLENIKQSESEINHVQDHALPKENIIDKLDNQKEHIDQSQHNINVLQENNINNHQLEPQEKPNIESFEPKNIDSEIILPENVETEEIIDDVPSPKHSNHETFEEETSESEHEEAVSEKNAHETVEHEETVSQESNPEKADNDGNVSQNSNNELNENEFVESEKSEHEAENLYFQGRSDEDSTHKDRNKKRRRKKRNNNKSKRKRKRKSKHDDYYDDNENEDSRKENDKEEESYYNKKKNLYLEKKNKKFKHNFIYLKNLFKENICINTLNVSNFISVSKDKLTATYTAWGKHTDIACVQVNKCALRDCSIYYFEVEILNCTNFSKIVIGMTNKNYTINKNPGSEYNSFGYKNDDGKKIIDGKIENYCNGYAKNDIIGCGINYFDNSAFFTKNGKYLGKACTINFKYDYYATVGLSTLGDRIKFHLNNFCFDIYNMIYEESEKERKIIKSIYVQKDIFSDIIKSHLIKCGYFNTYKSFMNYLEKNKYVDDNNSIESSTNYNNGMDGNIFPNKSNTSNNSQKKVENSSNNKNEKEKNKKNNTNDMNKDEKVEMEDDNNKITEGNHVKMEKEEKKKNKNDINLNIKNNTMKNLTEQNPHKSATSEQLDVTPEDNKKSG*HHHHHH*- |
| R0.1143700 | **MKFNKKRVAIATFIALIFVSFFTISSIQDAQAAE**RSTSENRNKRIGGPKLRGNVTSNIKFPSDNKGKIIRGSNDKLNKNSEDVLEQSEKSLVSENVPSGLDIDDIPKESIFIQEDQEGQTHSELNPETSEHSKDLNNNGSKNESSDIISENNKSNKVQNHFESLSDLELLENSSQDNLDKDTISTEPFPNQKHKDLQQDLNDEPLEPFPTQIHKDYKEKNLINEEDSEPFPRQKHKKVDNHNEEKNVFHENGSANGNQGSLKLKSFDEHLKDEKIENEPLVHENLSIPNDPIEQILNQPEQETNIQEQLYNEKQNVEEKQNSQIPSLDLKEPTNEDILPNHNPLENIKQSESEINHVQDHALPKENIIDKLDNQKEHIDQSQHNINVLQENNINNHQLEPQEKPNIESFEPKNIDSEIILPENVETEEIIDDVPSPKHSNHETFEEETSESEHEEAVSEKNAHETVEHEETVSQESNPEKADNDGNVSQNSNNELNENEFVESEKSEHEAENLYFQGRSKVKIGSFENFLERLNNIKEEDIIINDENTEEMFSSFLITFYKENEGRYTIQEQEYINNLLNILINKIRDNKNGRNFFSNLLCIHFKSITPYFINVKLKDSG*HHHHHH*- |
| R0.1314500 | **MKFNKKRVAIATFIALIFVSFFTISSIQDAQAAE**RSTSENRNKRIGGPKLRGNVTSNIKFPSDNKGKIIRGSNDKLNKNSEDVLEQSEKSLVSENVPSGLDIDDIPKESIFIQEDQEGQTHSELNPETSEHSKDLNNNGSKNESSDIISENNKSNKVQNHFESLSDLELLENSSQDNLDKDTISTEPFPNQKHKDLQQDLNDEPLEPFPTQIHKDYKEKNLINEEDSEPFPRQKHKKVDNHNEEKNVFHENGSANGNQGSLKLKSFDEHLKDEKIENEPLVHENLSIPNDPIEQILNQPEQETNIQEQLYNEKQNVEEKQNSQIPSLDLKEPTNEDILPNHNPLENIKQSESEINHVQDHALPKENIIDKLDNQKEHIDQSQHNINVLQENNINNHQLEPQEKPNIESFEPKNIDSEIILPENVETEEIIDDVPSPKHSNHETFEEETSESEHEEAVSEKNAHETVEHEETVSQESNPEKADNDGNVSQNSNNELNENEFVESEKSEHEAENLYFQGRSGPYEKDCIQLKPEKNNMIVGSYEFMDRKASCIISIFNRSDKKKEPVFKSTKIQDKFEIQVPAAAVYSFCYDNRKNSDVTIMFTLRVKESHNVNDSELSTIDDVKQINEKTSELFDQFLEVFDEQERMMEKSDLYKQFNEKMNSKSG*HHHHHH*- |
| R0.1348000 | **MKFNKKRVAIATFIALIFVSFFTISSIQDAQAAE**RSTSENRNKRIGGPKLRGNVTSNIKFPSDNKGKIIRGSNDKLNKNSEDVLEQSEKSLVSENVPSGLDIDDIPKESIFIQEDQEGQTHSELNPETSEHSKDLNNNGSKNESSDIISENNKSNKVQNHFESLSDLELLENSSQDNLDKDTISTEPFPNQKHKDLQQDLNDEPLEPFPTQIHKDYKEKNLINEEDSEPFPRQKHKKVDNHNEEKNVFHENGSANGNQGSLKLKSFDEHLKDEKIENEPLVHENLSIPNDPIEQILNQPEQETNIQEQLYNEKQNVEEKQNSQIPSLDLKEPTNEDILPNHNPLENIKQSESEINHVQDHALPKENIIDKLDNQKEHIDQSQHNINVLQENNINNHQLEPQEKPNIESFEPKNIDSEIILPENVETEEIIDDVPSPKHSNHETFEEETSESEHEEAVSEKNAHETVEHEETVSQESNPEKADNDGNVSQNSNNELNENEFVESEKSEHEAENLYFQGRSSLNGVDLHTTDFINEKEEWMKKYENIELEYGALKKKFEEFSIDYKNKNEEIKNFATIKKDMILRYAYMKNENEFLKTQLNILILEKEEEEIKKRELIEINEKQKREIDGYKSG*HHHHHH*- |
| R0.1433200B | **MKFNKKRVAIATFIALIFVSFFTISSIQDAQAAE**RSTSENRNKRIGGPKLRGNVTSNIKFPSDNKGKIIRGSNDKLNKNSEDVLEQSEKSLVSENVPSGLDIDDIPKESIFIQEDQEGQTHSELNPETSEHSKDLNNNGSKNESSDIISENNKSNKVQNHFESLSDLELLENSSQDNLDKDTISTEPFPNQKHKDLQQDLNDEPLEPFPTQIHKDYKEKNLINEEDSEPFPRQKHKKVDNHNEEKNVFHENGSANGNQGSLKLKSFDEHLKDEKIENEPLVHENLSIPNDPIEQILNQPEQETNIQEQLYNEKQNVEEKQNSQIPSLDLKEPTNEDILPNHNPLENIKQSESEINHVQDHALPKENIIDKLDNQKEHIDQSQHNINVLQENNINNHQLEPQEKPNIESFEPKNIDSEIILPENVETEEIIDDVPSPKHSNHETFEEETSESEHEEAVSEKNAHETVEHEETVSQESNPEKADNDGNVSQNSNNELNENEFVESEKSEHEAENLYFQGRSTSDISSTTTLTYKAVENNGDVKNNEHIIKESIQEKQDNNDAIFHHHQVFQKDTECACDENGIWNNTESKILNTNCQCEKNENNIKDIKKKDNINESNIKEDKKEEYFLSNQLNTFINFLLLLWGINISFDHINHEEFLITEDLSTDTDDNFINVDTNYSTNKFTTTTTTNYNNSNNYKDDDEKLKCINQTNKNVKCKKEDIRNNYSPYDTNDEDKEEKITNTWKMKTQNLYKKNYQSESNMNISIKKYRKNTQKKNKNEERTKSLIIINRKGNNEKKKNKNKINNNNYNKINKNLLYHNTDQQNINTFDDNQNYVKNQEYLNSG*HHHHHH*- |

**Bold:** secretion signal; Underlined: linkers; *Italic:* 6x histidine tag; Grey: GLURP.R0; Cyan: TEV protease site

**Supplementary Table 3:** Expression screening by ELISA (anti-his) for proteins expressed in *Lactococcus lactis.*

| **Gene ID** | **Colony 1** | **Colony 2** | **Colony 3** | **Colony 4** | **Colony 5** |
| --- | --- | --- | --- | --- | --- |
| PF3D7_0305300* | 2.00 | 2.50 | 2.60 | 2.00 | 2.00 |
| PF3D7_1014300 | 0.04 | 0.05 | 0.04 | 0.04 | 0.04 |
| PF3D7_1021100 | 0.05 | 0.05 | 0.04 | 0.05 | 0.04 |
| PF3D7_1107900* | 0.82 | 0.14 | 1.18 | 0.65 | 0.17 |
| PF3D7_1143700* | 0.20 | 0.21 | 0.04 | 0.13 | 0.06 |
| PF3D7_1306500B | 0.05 | 0.04 | 0.04 | 0.04 | 0.05 |
| PF3D7_1306500C* | 1.01 | 1.18 | 1.25 | 1.44 | 0.10 |
| PF3D7_1314500* | 1.50 | 1.16 | 1.42 | 1.23 | 0.84 |
| PF3D7_1324600A | 0.05 | 0.05 | 0.05 | 0.05 | 0.05 |
| PF3D7_1324600B | 0.05 | 0.05 | 0.05 | 0.05 | 0.04 |
| PF3D7_1348000* | 1.12 | 1.02 | 0.96 | 1.14 | 1.28 |
| PF3D7_1360500A* | 0.86 | 0.19 | 0.17 | 0.32 | 0.47 |
| PF3D7_1360500B | 0.04 | 0.04 | 0.04 | 0.04 | 0.04 |
| PF3D7_1433200A | 0.05 | 0.05 | 0.05 | 0.05 | 0.05 |
| PF3D7_1433200B | 0.05 | 0.05 | 0.05 | 0.05 | 0.05 |
| PF3D7_1433200C | 0.05 | 0.05 | 0.05 | 0.04 | 0.04 |
| PF3D7_1449000 | 0.04 | 0.04 | 0.05 | 0.05 | 0.06 |

Data are presented as raw optical density values as an average of two duplicate wells

*These antigens were selected for large scale fermentation

**Supplementary Table 4:** Expression screening by ELISA (anti-his) for R0 fusion proteins expressed in *Lactococcus lactis*.

| **Gene ID** | **Colony 1** | **Colony 2** | **Colony 3** | **Colony 4** | **Colony 5** |
| --- | --- | --- | --- | --- | --- |
| PF3D7_0305300* | 1.26 | 1.41 | 1.91 | 1.71 | 2.00 |
| PF3D7_1014300* | 2.36 | 2.42 | 2.42 |  |  |
| PF3D7_1021100 | 0.03 | 0.04 | 0.01 | 0.02 | 0.01 |
| PF3D7_1107900* | 0.24 | 0.17 | 0.31 | 0.35 | 0.34 |
| PF3D7_1143700* | 2.34 | 2.36 | 2.36 |  |  |
| PF3D7_1306500A | 0.60 | 0.79 | 0.48 | 0.85 | 0.52 |
| PF3D7_1306500B | 0.18 | 0.49 | 0.47 |  |  |
| PF3D7_1314500* | 2.52 | 2.67 | 2.58 |  |  |
| PF3D7_1324600A* | 0.18 | 0.19 | 0.14 | 0.17 | 0.11 |
| PF3D7_1324600B | 0.08 | 0.07 | 0.05 |  |  |
| PF3D7_1348000* | 2.38 | 2.45 | 2.47 |  |  |
| PF3D7_1360500A | 0.14 | 0.14 | 0.11 |  |  |
| PF3D7_1360500B | 0.10 | 0.09 | 0.13 |  |  |
| PF3D7_1433200A* | 0.24 | 0.17 | 0.31 | 0.35 | 0.34 |
| PF3D7_1433200B* | 0.59 | 2.04 | 1.87 |  |  |
| PF3D7_1433200C | 0.07 | 0.07 | 0.05 |  |  |
| PF3D7_1449000 | 0.08 | 0.06 | 0.05 |  |  |

Individual colonies (1-5) were screened for protein expression. Data are presented as raw optical density values as an average of two duplicate wells

*These antigens were selected for large scale fermentation

We did not attempt expression of 1306500C (TBC2), because this antigen was already successfully produced in S2 cells and *L. lactis*.

**Supplementary Table 5:** Raw data from the standard membrane feeding assay.

| **Immunization group** | **Number of dissected mosquitoes** | **Percentage of mosquitoes infected** | **Total number of oocysts** | **Average oocysts/mosquito** | **Percentage TRA** |
| --- | --- | --- | --- | --- | --- |
| TBC1^DM^ | 20 | 100 | 290 | 14.5 | 8 |
| TBC1^LL*^ | 20 | 95 | 363 | 18.2 | -15 |
| TBC2^DM^ | 20 | 100 | 320 | 16.0 | -2 |
| TBC2^LL^ | 22 | 95 | 271 | 12.3 | 22 |
| TBC3^DM^ | 20 | 100 | 367 | 18.4 | -17 |
| TBC3^LL^ | 20 | 100 | 318 | 15.9 | -1 |
| TBC3^LL*^ | 20 | 100 | 351 | 17.6 | -12 |
| TBC4^LL^ | 20 | 100 | 296 | 14.8 | 6 |
| TBC5^LL*^ | 20 | 100 | 337 | 16.9 | -7 |
| TBC6^LL*^ | 20 | 90 | 276 | 13.8 | 12 |
| TBC7^LL*^ | 17 | 100 | 223 | 13.1 | 17 |
| R0.6C | 20 | 35 | 16 | 0.8 | 95 |
| Serum control | 40 | 100 | 629 | 15.7 |  |
| mAb 2A2 control | 20 | 5 | 1 | 0.1 | 100 |

mAb 2A2 is an α-Pfs230 monoclonal antibody; TRA: Transmission reducing activity.

*indicate antigens that were expressed as R0 chimeras.
